# Genome-Wide Identification of the CrRLK1L Subfamily and Comparative Analysis of Its Role in the Legume-Rhizobia Symbiosis

**DOI:** 10.3390/genes11070793

**Published:** 2020-07-14

**Authors:** Jorge Solis-Miranda, Citlali Fonseca-García, Noreide Nava, Ronal Pacheco, Carmen Quinto

**Affiliations:** Departamento de Biología Molecular de Plantas, Instituto de Biotecnología, Universidad Nacional Autónoma de México, Avenida Universidad 2001, Colonia Chamilpa, Cuernavaca, Morelos 62210, Mexico; jsolis@ibt.unam.mx (J.S.-M.); fonsecac@ibt.unam.mx (C.F.-G.); noreide@ibt.unam.mx (N.N.); ronal.pacheco@mail.ibt.unam.mx (R.P.)

**Keywords:** CrRLK1L, expression profile, legumes, nodule symbiosis, phylogenetic analysis

## Abstract

The plant receptor-like-kinase subfamily CrRLK1L has been widely studied, and CrRLK1Ls have been described as crucial regulators in many processes in *Arabidopsis thaliana* (L.), Heynh. Little is known, however, about the functions of these proteins in other plant species, including potential roles in symbiotic nodulation. We performed a phylogenetic analysis of CrRLK1L subfamily receptors of 57 different plant species and identified 1050 CrRLK1L proteins, clustered into 11 clades. This analysis revealed that the CrRLK1L subfamily probably arose in plants during the transition from chlorophytes to embryophytes and has undergone several duplication events during its evolution. Among the *CrRLK1Ls* of legumes and *A. thaliana*, protein structure, gene structure, and expression patterns were highly conserved. Some legume *CrRLK1L* genes were active in nodules. A detailed analysis of eight nodule-expressed genes in *Phaseolus vulgaris* L. showed that these genes were differentially expressed in roots at different stages of the symbiotic process. These data suggest that *CrRLK1Ls* are both conserved and underwent diversification in a wide group of plants, and shed light on the roles of these genes in legume–rhizobia symbiosis.

## 1. Introduction

Plants are continually exposed to many environmental conditions that they must contend with to survive. These conditions are perceived by plant cells as physical or chemical signals that are sensed by plasma membrane receptors. The receptor-like kinase (RLK) family is one of the largest receptor families and is represented in all organisms. RLKs are involved in many processes, including the perception of pathogens and symbiotic partners. Defense-associated RLKs are activated by pathogen-derived molecules (such as flagellin or fungal chitin) and initiate defense responses. Other specific RLKs bind to signal molecules from mycorrhizal fungi or rhizobia, triggering symbiosis.

In the model plant *Arabidopsis thaliana* (L.), Heynh, about 600 RLKs have been described, and they have been classified into several subfamilies [1]. The *Catharanthus roseous* (L.), D.Don RLK-1L (CrRLK1L) subfamily is unique to plants [2] and has been widely studied in *A. thaliana*. CrRLK1L receptors are characterized by a carbohydrate-binding domain known as the malectin-like domain for its similarity to the animal protein malectin [3]. The *A. thaliana* genome harbors 17 CrRLK1Ls [2], of which FERONIA (FER) has been the most studied. FER was initially characterized as a regulator of female fertility; later, it was described as an important regulator in some phytohormone signaling pathways [4,5,6,7,8,9] and was shown to be essential for polar growth in root hair cells (Table S1) [10]. More recently, FER has been reported to be a negative regulator of the immune response in plants [11,12], an activator of protein synthesis [13], and a regulator of growth in response to metabolic status (the C/N ratio) (Table 1) [14].

In association with FER, other CrRLK1Ls, such as HERKULES1 (HERK1), HERK2, and THESEUS1 (THE1), are involved in cell wall maintenance and cytoplasmic membrane homeostasis (Table 1) [4]. During fertilization, two CrRLK1Ls, HERK1 and ANJEA, together with FER, mediate male–female gametophyte interaction at the synergid cells (Table 1) [33]. Four other CrRLK1Ls, ANXUR1 (ANX1), ANX2, BUDDHAs PAPER SEAL1 (BUPS1), and BUPS2 are essential for preserving the integrity of the pollen tube during growth (Table 1) [30,31]. The CrRLK1L CAP regulates calcium-dependent pollen tube growth, and is also implicated in maintaining cell wall composition in root hairs during tip growth (Table 1) [35,36]. CURVY1, another CrRLK1L, is important in trichome and tapetal cell morphogenesis, the vegetative-to-reproductive state transition, and seed production (Table 1) [37]. Four other CrRLK1Ls, MEDOS1-4, are associated with the regulation of plant development in response to the presence of metal ions (Table 1) [38].

RALF (Rapid alkalinization factor) peptides have been described as ligands of some of the CrRLK1L receptors [8,20,39,40]. These peptides are widely distributed in all land plants, and their activity is associated with pH modulation and the production of reactive oxygen species (ROS) [39,41,42]. *A. thaliana* has 34 RALF peptides, which are differentially expressed in different plant tissues [43,44], and a total of 795 RALFs have been identified in 51 different plant species (monocots, eudicots, and early-diverging lineages) [44]. The cysteine-rich peptide RALF1 was the first peptide described as a FER ligand [39]. The RALF1-FER complex is important for fine-tuning the plant response to non-peptide hormones, root elongation, and polar root hair growth in *A. thaliana* [8]. RALF34, RALF4, and RALF19 interact with the CrRLK1L complex BUPS1/2-ANX1/2 during fertilization [40]. RALF34 also binds to THE1 in roots, a signaling step required for division of the pericycle during lateral root initiation [40]. RALF23 acts as a negative regulator of immunity through its interaction with FER [20]. In symbiotic interactions, it has been reported that the *Medicago truncatula* Gaertn. homolog of RALF1 (MtRALF1) functions as a negative regulator of nodule formation during the development of nitrogen-fixing symbioses; however, the receptor that recognizes MtRALF1 and triggers this inhibition of nodule formation is unknown [45].

The formation of nitrogen-fixing root nodules is a complex process and occurs almost exclusively in legumes, a large family of plants [46]. In this process, the plant roots interact with the Gram-negative soil bacteria, known as rhizobia, which through a molecular dialogue between these two partners, induce the formation of a new structure, the nodule, where the rhizobia gain the ability to fix atmospheric nitrogen. Symbiotic development includes changes in gene expression, suppression of defense mechanisms, induction of root cell division, and formation of nitrogen-fixing nodules. Because this is an expensive process for the plant, the establishment of symbiosis is highly regulated. Inhibition of nitrogen fixation, inhibition of symbiosis when interacting with incompatible or non-fixing bacteria, and control of the number of nodules are three of the essential regulatory mechanisms that match the degree of nodulation to the needs of the plant [47].

Although most of the CrRLK1Ls have been studied in *A. thaliana*, little is known about these proteins in other plant models, including legumes. Therefore, our knowledge is very limited about the function of CrRLK1Ls during the legume-rhizobia symbiosis. To address this gap, we first performed a robust phylogenetic analysis of the CrRLK1L subfamily members of more than 60 plant species, including four species of legumes. We compared the gene features and expression profiles of CrRLK1Ls between different organs in four legumes and *A. thaliana* and demonstrated that some *CrRLK1L* genes are expressed in legume nodules. Among these are eight genes that were differentially expressed over the course of nodule development in *P. vulgaris* roots inoculated with rhizobia. This study provides a robust and comprehensive phylogenetic analysis of the CrRLK1L subfamily and unveils, for the first time, relevant information about the presumed role of this receptor subfamily in legume–rhizobia symbiosis.

## 2. Materials and Methods

### 2.1. Identification of CrRLK1 Subfamily Proteins in 62 Plant Species

To identify all CrRLK1L proteins in 61 plant genomes available in the Phytozome v12.1 database (https://phytozome.jgi.doe.gov) [48] and in the *Lotus japonicus* L. genome (https://lotus.au.dk/) [49], BLASTP searches using the *A. thaliana* CrRLK1L protein sequences as query were performed. In both databases, default settings for e-values (e^−1^ value) and the number of hit sequences (100 hits) were used. To confirm that the sequences were part of the CrRLK1L family, they were analyzed with Pfam 32.0 (http://pfam.xfam.org) [50] and filtered by the presence of the characteristic malectin-like and kinase domains in this subfamily. A total of 1050 proteins sequences were confirmed as CrRLK1L proteins and downloaded from both databases.

### 2.2. Phylogenetic Analysis of the CrRLK1L Subfamily

All 1050 protein sequences were aligned using the MUSCLE algorithm [51] followed by a manual optimization of the misaligned sequences in the AliView editor [52]. An approximately maximum-likelihood phylogenetic tree [53] was created for edited sequence alignment with IQ-TREE 1.6.12 [54], using the JTT+F+R10 substitution model with 1000 bootstraps and default parameters. The Pearson correlation coefficient was calculated to determine the relation between the number of *CrRLK1L* genes and the genome size or the number of *CrRLK1L* genes between the total number of genes, for the analyzed species.

To explore the possibility that CrRLK1Ls participate in legume–rhizobia symbiosis, a phylogenetic analysis of the CrRLK1L protein sequences of *P. vulgaris*, *L. japonicus*, *Glycine max* (L.), Merr. and *M. truncatula* was performed. To compare legumes with other model plants, we selected *A. thaliana* as the model plant in which the *CrRLK1Ls* genes have been more studied, and *Physcomitrella patens* (Hedw.) Bruch & Schimp as a representative moss species. All of these plant species have complete accurate genome and proteome annotations in the Phytozome and Lotus Base databases, as well as available expression profile data. Alignment of CrRLK1L protein sequences from these six species was also done using the MUSCLE algorithm [51] within the AliView alignment editor [52], and a manual optimization of the misaligned regions. Then, an approximately maximum-likelihood phylogenetic unrooted tree [53] was established for full-length aligned protein sequences with IQ-TREE 1.6.12 [54] with a JTT+F+R7 substitution model and 1000 bootstraps for reliability, using the default parameters. The clades and subclades of both phylogenetic trees were analyzed using MEGA7 [55].

### 2.3. Analysis of CrRLK1L Protein Motif Conservation in Legumes and A. thaliana

Protein motif conservation of the 150 CrRLK1Ls present in *A. thaliana* and in the four legumes analyzed were determined using the conserved sequence motif analyzer MEME (http://meme-suite.org) [56]. The analysis was done using the full-length amino acid sequences, setting the maximum number to 15 motifs, the number of expected motifs to any number of repetitions, and the length of the motif to 10–200 amino acids. The other parameters were kept as default. To calculate the theoretical molecular weight and isoelectric point, the 150 proteins sequences were submitted to the ExPASy web server (https://web.expasy.org/compute_pi/) [57].

### 2.4. Gene Structure, Chromosomal Localization, and Synteny Analysis of the CrRLK1L Gene Subfamily of Legumes, A. thaliana, and Sorgum bicolor (L.), Moench

The gene structure and chromosomal localization data of the 33 *P. vulgaris*, 18 *L. japonicus*, 46 *G. max*, 36 *M. truncatula*, 17 *A. thaliana*, and 14 *S. bicolor CrRLK1L* genes were retrieved from the Phytozome v12.1 [48] database and Lotus Base [49]. *S. bicolor* was used to evaluate the differences between eudicot and monocot *CrRLK1L* genes features, since it is a monocot model with complete genome sequence and gene expression information. The gene structure map for each species was represented using the free resource Gene Structure Display Server 2.0 (http://gsds.gao-lab.org/Gsds_about.php). For the chromosome distribution, data were uploaded into the free resource PhenoGram Plot (http://visualization.ritchielab.org/phenograms/plot) [58]. For synteny analysis, the protein sequences and annotation files of the full genomes of *P. vulgaris*, *L. japonicus*, *G. max*, and *L. japonicus* were downloaded from the previously mentioned databases [48,49]. For each case, an m8 format BLASTP file and a simplified gff file were used as inputs to the collinearity scanner toolkit MCScanx (http://chibba.pgml.uga.edu/mcscan2/) [59] to determine synteny between *CrRLK1L* genes in the legume species and to compare it with that in *A. thaliana*.

### 2.5. In Silico Analysis of the CrRLK1L Gene Family Expression in Legumes, A. thaliana, and P. patens

Expression profiles of the 33 members of the *P. vulgaris CrRLK1L* gene subfamily were retrieved from the Common bean Gene Expression Atlas, PvGEA (https://plantgrn.noble.org/PvGEA/) [60]. *L. japonicus* expression profile data were downloaded from the *L. japonicus* reference genome transcript explorer in Lotus Base [49]. *M. truncatula* expression profile data were downloaded from the *M. truncatula* Gene Expression Atlas (MtGEA) [61,62] by BLASTN. The expression profiles of the 46 *G. max*, 17 *A. thaliana*, and 6 *P. patens CrRLK1L* genes were obtained from the Bio-Analytic Resource for Plant Biology (BAR) [61,63,64,65]. The distribution and abundance of the expression profile of the genes were presented in heatmaps with the function heatmap.2 of the gplot package [66] using R. To identify the shared genes expressed in nodules of *P. vulgaris*, *L. japonicus*, and *G. max*, a Venn diagram was drawn using the Venn diagram drawing tool (http://bioinformatics.psb.ugent.be/webtools/Venn/).

### 2.6. Plant Growth Conditions and RT-qPCR Assays

Common bean (*P. vulgaris cv*. Negro Jamapa) seeds were surface-sterilized and germinated for 2 days (dpg) at 28 °C in darkness. For RT-qPCR accumulation profile analysis during nodulation, 2 dpg seedlings were transplanted into pots with vermiculite and inoculated with *Rhizobium tropici* CIAT899 at an OD_600_ of 0.05, or only with Fahraeus media as mock. Roots were harvested at 5, 7, 14, and 21 days post-inoculation (dpi). The tissues selected for RT-qPCR were immediately frozen in liquid nitrogen and stored at −70°C until RNA extraction. RNA was isolated from the frozen tissues using Trizol reagent (Sigma-Aldrich, St. Louis, MO, USA), following the manufacturer’s instructions. RNA integrity was verified by electrophoresis and the concentration was assessed using a NanoDrop2000 spectrophotometer (Thermo Fisher Scientific, Waltham, MA, USA). To eliminate DNA contamination, the RNA samples were incubated with RNase-free DNase (1 U/µL; Roche, Basel, Switzerland).

Complementary DNA (cDNA) was synthesized using Thermo Scientific RevertAid Reverse Transcriptase (200 U/µL, Thermo Scientific, Waltham, MA, USA), with 200 ng of DNA-free RNA as template and following the manufacturer’s instructions. RT-qPCR assays were performed using Maxima SYBR Green/ROX qPCR Master Mix (2X) (Thermo Scientific, Waltham, MA, USA), in a real time PCR system (QuantStudio 5; Applied Biosystems, Waltham, MA, USA) with the following thermal cycle: 95 °C for 10 min, 30 cycles of 95 °C for 15 s, and 60 °C for 60 s. Experiments were normalized with the reference gene *Elongation Factor 1α* (*EF1 α*) [67]. Relative expression values were calculated using the formula 2^−ΔCt^, where the cycle threshold value ΔCt is equal to the Ct of the gene of interest minus the Ct of the reference gene. Three biological replicates with three technical repeats were performed for each dataset. The gene-specific oligonucleotides used in this study are listed in Supplementary Materials Table S1.

## 3. Results

### 3.1. Identification and Phylogenetic Analysis of CrRLK1L Proteins in Diverse Plant Species

Previous reports identifying CrRLK1L subfamily receptors have focused on a few model species, such as *A. thaliana* and *Oryza sativa* L. To study the potential function of this receptor subfamily in legume-rhizobia symbiosis, we expanded our analysis of these proteins to other plant species by searching for CrRLK1Ls in 61 plant genomes deposited in Phytozome v12.1 and also in the *L. japonicus* genome. We searched for CrRLK1L homologs in the 62 species, followed by a domain analysis of the identified proteins to confirm the presence of the characteristic malectin-like and kinase domains of the CrRLK1L subfamily. We identified a total of 1050 CrRLK1L proteins in 57 of the 62 species analyzed. None of the five chlorophyte genomes we searched had a significant hit related to the CrRLK1L subfamily. By contrast, at least one CrRLK1L was encoded in every land plant genome in our analysis (Table S2). The complete data are summarized in Table S2, and the IDs of the 1050 CrRLK1L proteins are listed in Table S3.

**Figure 1:**
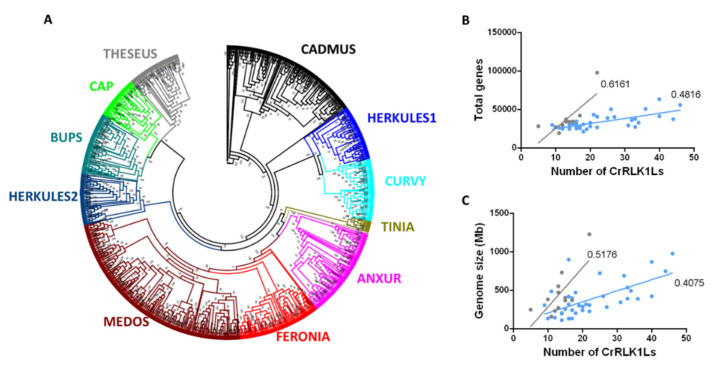
Phylogenetic relationship among 1050 CrRLK1L proteins and the relationship between *CrRLK1L* number versus gene size and gene number. (**A**) Unrooted approximately maximum-likelihood phylogenetic tree inferred from the 1050 CrRLK1L proteins present in 57 plant species. The clades, indicated in different colors, are named based on the *A. thaliana* CrRLK1L names. The CADMUS clade contains uncharacterized CrRLK1Ls from *A. thaliana*, and the TINIA clade corresponds to a clade formed only with the CrRLK1L proteins of *S. moellendorffii*, *S. fallax*, *P. patens*, and *M. polymorpha*. The phylogenetic tree was constructed using IQ-TREE software with the JTT+F+R10 substitution model with 1000 bootstrap iterations. (**B**) Relationship between total number of genes within the genome and number of *CrRLK1L* genes for monocotyledons (gray) and eudicots (blue). (**C**) Relationship between genome size and number of *CrRLK1L* genes for monocotyledons (gray) and eudicots (blue).

Based on a phylogenetic analysis of the amino acid sequences of these 1050 proteins, we established that they are distributed into 11 clades (Figure 1A, Figure S1). One of these clades consisted exclusively of CrRLK1Ls of the most ancient plant species included in this analysis: three bryophytes (*Marchantia polymorpha* L., *P. patens*, and *Sphagnum fallax* (H.Klinggr) H.Klinggr) and a clubmoss (*Selaginella moellendorffii* Hieron). This clade was named TINIA (after the first of the Etruscan gods and father of Herkules), following the mythological nomenclature used for other CrRLK1L clades (Figure 1A, Figure S1). Of the remaining ten clades, nine were named according to the nomenclature employed for the *A. thaliana* protein belonging to each clade (Figure 1A, Figure S1). A clade carrying the two uncharacterized *A. thaliana* CrRLK1Ls was named CADMUS (after the Etruscan king founder of Thebes) (Figure 1A, Figure S1). Eudicots had an average of 22 CrRLK1L proteins, whereas monocots had fewer, with an average of 13 (Figure 1A, Figure S1, Table S1). This difference in the number of CrRLK1Ls could be associated with the greater size of eudicot genomes compared to those of monocots, since there is a moderate correlation between the number of CrRLK1Ls and the genome size and the number of total genes in both groups (eudicots, *r* = 0.55 and *r* = 0.47, respectively; monocots, *r* = 0.51 and *r* = 0.62, respectively) (Figure 1B,C). These data suggest that the CrRLK1L subfamily probably appeared during the transition from chlorophytes to embryophytes, and that the number of members increased along with the size of the genome and the total number of genes during evolution.

### 3.2. Phylogenetic Analysis of the CrRLK1L Subfamily in Legumes, A. thaliana, and P. patens

Legumes have the ability to establish a symbiotic relationship with rhizobia and form nitrogen-fixing nodules. To explore the possibility that CrRLK1Ls participate in legume–rhizobia symbiosis, we constructed a phylogenetic tree that included all the CrRLK1Ls of four model legumes (*L. japonicus*, *M. truncatula*, *G. max,* and *P. vulgaris*), *A. thaliana*, and the moss *P. patens*.

As expected, all five *P. patens* proteins were placed in the basal TINIA clade, separated from the proteins of the four legumes and *A. thaliana* (Figure S2). The remaining CrRLK1L proteins were distributed among ten clades, each containing at least one CrRLK1L from *A. thaliana* and one to several proteins from the legumes. Although no clade was confined exclusively to legumes, some clades had more members in the legumes than are present in *A. thaliana*. In this context, the MEDOS clade was particularly interesting because *P. vulgaris*, *M. truncatula*, and *G. max* each have a relatively large number of these proteins (16, 18, and 24, respectively), whereas *A. thaliana* has only four (Table 2). These observations indicate that although there is no group of proteins exclusively associated with legumes, there is at least a four-fold increase in the number of CrRLK1L proteins in this plant family compared to *A. thaliana*, particularly in the MEDOS clade.

### 3.3. Features of the CrRLK1L Subfamily Proteins in Legumes and A. thaliana

Since we identified no CrRLK1L clade that was exclusive to legumes, we wondered whether some of these proteins, which have important functions in other plant processes, could have been recruited to function in legume-rhizobia symbiosis. To assess this possibility, we analyzed the molecular characteristics of the CrRLK1L proteins of the four legumes previously examined and *A. thaliana*. There are 33 CrRLK1L proteins encoded in the *P. vulgaris* genome, whereas in *A. thaliana* there are 17, in both cases distributed among ten different clades. *M. truncatula*, *G. max*, and *L. japonicus* have 36, 46, and 18 CrRLK1Ls, respectively, scattered among nine clades (Figure S2).

**Figure 2:**
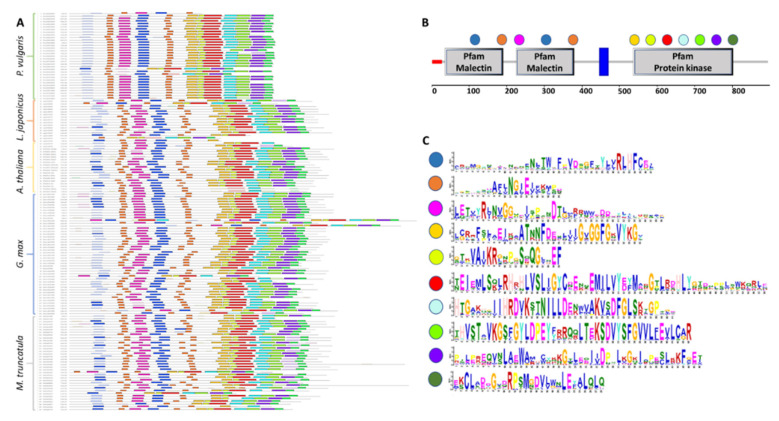
CrRLK1L protein sequence conservation and characteristic motifs in legumes and *A. thaliana*. MEME was used to identify motifs in the 150 CrRLK1Ls from four legumes and *A. thaliana*. (**A**) Diagram of the motifs in the CrRLK1L protein sequences from *P. vulgaris*, *L. japonicus*, *G. max*, *M. truncatula*, and *A. thaliana*. Significant overrepresented motifs are graphically depicted by bars corresponding to their predicted position. (**B**) Localization of overrepresented motifs identified using MEME in the CrRLK1L protein domains. (**C**) Logo of the overrepresented motifs identified with MEME; the color code corresponds with that used in (**A**,**B**).

The CrRLK1L proteins are defined by the presence of a malectin-like domain in the amino-terminal region and a kinase domain in the carboxy-terminal region. To characterize these conserved motifs, the CrRLK1L sequences of the four legumes under study and *A. thaliana* were examined using MEME software (Figure 2). In the 150 sequences analyzed, ten different motifs were identified; seven of these were located in the kinase domain, and only three in the malectin domain, two of them duplicated. The motifs located in the kinase domain were longer and more conserved than those in the malectin-like domain (Figure 2). No additional features were observed that could be associated with a given species or phylogenetic clade, beyond the particularities of individual proteins, such as shorter or longer amino acid sequences.

The 33 *P. vulgaris* CrRLK1L proteins ranged from 450 to 899 amino acids (aa) in length and 50.59 to 99.43 kDa in molecular weight (MW) (Table 2). The theoretical isoelectric point (iP) of most of the common bean proteins is slightly acidic (4.83 to 6.67), though seven proteins are slightly alkaline (7.05 to 8.67) (Table 2). The *L. japonicus* CrRLK1Ls showed similar features to those of common bean, with MWs of 57.31 to 97.76 kDa and lengths of 513 to 891 aa. Furthermore, most of the *L. japonicus* proteins have acidic iPs (5.93 to 6.98), whereas only three of them have alkaline iPs (7.59 to 8.87) (Table 2). The CrRLK1L proteins have a broader MW range in *M. truncatula* and *G. max* (64.53 to 130.78 and 72.73 to 133.95 kDa, respectively) and are longer (568 to 1152 aa and 647 to 1186 aa, respectively) than to those of *P. vulgaris* (Table 2). In *M. truncatula*, only five proteins have alkaline iPs (7.05 to 8.81), whereas the remaining 31 have acidic iPs (5.21 to 6.91) (Table 2). In *G. max*, eight of the proteins were alkaline (7.04 to 8.81) and 38 are acidic (5.24 to 6.55) (Table 2). In comparison to the legume proteins, the *A. thaliana* CrRLK1Ls have narrower ranges of MW (90.68 to 98.16 kDa) and length (806 to 895 aa). Only one *A. thaliana* protein has an alkaline iP (7.6), whereas 16 have a slightly acidic iP (5.51 to 6.54) (Table 2).

Despite high conservation of the CrRLK1L domains, there were some physicochemical differences between the legume proteins we studied and those of *A. thaliana*. This variation could be associated with the higher number of proteins observed in *G. max*, *P. vulgaris*, and *M. truncatula* compared to *A. thaliana*, which could have allowed more divergence of the proteins over time.

### 3.4. Chromosomal Localization and Synteny of CrRLK1L Genes in Legumes and A. thaliana

To compare the genome distributions of *CrRLK1L* genes in *A. thaliana* and in the four legumes under study, we used PhenoGram Plot to map the chromosome locations of the *CrRLK1L* genes in each plant species. The *P. vulgaris CrRLK1L* genes are distributed among seven of the 11 chromosomes, mainly on chromosomes four and eight (Figure S3A). The *P. vulgaris MEDOS* genes were mapped to chromosomes three (two genes), four (ten genes), and eight (four genes). *M. truncatula* and *G. max* have a similar gene distribution, the 46 *G. max CrRLK1L* genes are distributed among 14 of the 20 chromosomes, with two groups of MEDOS clade genes, one on chromosome 13 (six genes) and the other on chromosome 18 (eight genes) (Figure S3C). *M. truncatula* has 36 *CrRLK1L* genes, located on seven chromosomes, and two clusters of MEDOS clade genes on chromosomes five (four genes) and seven (12 genes) (Figure S3D). In the *L. japonicus* and *A. thaliana* genomes, *CrRLK1L* genes are distributed on five chromosomes, with only one small cluster, consisting of *MEDOS* genes, in each species, on chromosome two in *L. japonicus* (Figure S3B) and on chromosome five in *A. thaliana* (Figure S3E). Previously, we found that the MEDOS clade is absent in most monocots; therefore, we decided to also examine the distribution of the *CrRLK1L* genes in *S. bicolor.* In this species, all *CrRLK1L* genes were located on six chromosomes, and no clustering was observed (Figure S3F). These data suggest that there has been an expansion of the *MEDOS* genes in eudicots, and that some species have undergone greater expansion than others.

To further explore the evolutionary trajectories of the *CrRLK1L* genes, we evaluated the local synteny among the *CrRLK1L* genes in *P. vulgaris*, *G. max*, *L. japonicus*, *M. truncatula*, and *A. thaliana*. Chromosomal synteny was evaluated in these five species individually and also between species using MCScanx software. This analysis showed that four pairs of genes are syntenic in *P. vulgaris*, corresponding to 25% of the *CrRLK1L* genes. This was close to the percentage of gene synteny in the *P. vulgaris* genome overall (28.68%) (Figure S4, Table S4). In *L. japonicus*, only one pair of syntenic genes was identified, corresponding to 11% of *CrRLK1L* genes. Despite being lower than the *CrRLK1L* gene synteny in *P. vulgaris*, this percentage is above the median for the *L. japonicum* genome overall (4.74%) (Figure S4, Table S4). In *G. max*, 21 pairs of the *CrRLK1L* genes are syntenic, corresponding to 25 different genes (54.35% of the *CrRLK1L* genes). This gene synteny is slightly low given that 68.13% of all the genes in *G. max* genome are syntenic (Figure S4, Table S4). In *A. thaliana*, two pairs of genes have synteny, which corresponds to 23.53% of the *CrRLK1L*s, similar to the 27.1% synteny of the *A. thaliana* genome overall (Figure S4, Table S4).

**Figure 3:**
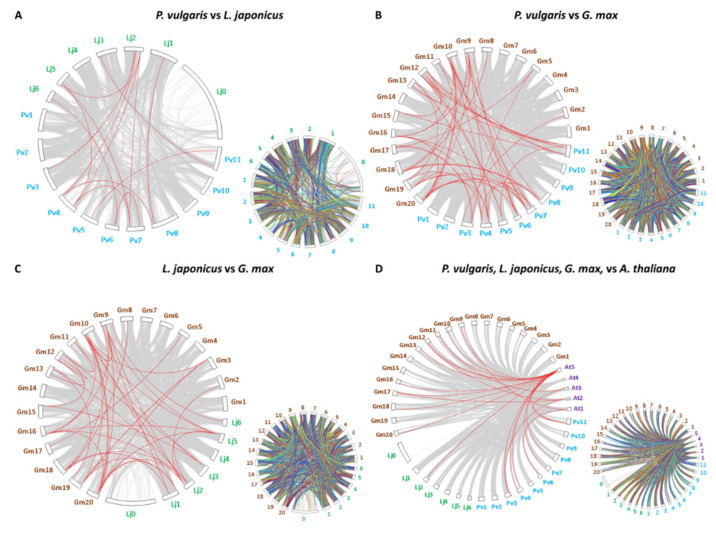
Synteny of the *CrRLK1L* genes in *P. vulgaris*, *L. japonicus*, *G. max*, and *A. thaliana*. MCScan software was used to analyze the syntenic correlation between species. (**A**) Synteny map of *CrRLK1Ls* between *L. japonicus* and *P. vulgaris.* (**B**) Synteny map of *CrRLK1Ls* between *G. max* and *P. vulgaris.* (**C**) Synteny map of *CrRLK1Ls* between *L. japonicus* and *G. max.* (**D**) Synteny map of *CrRLK1Ls* between *A. thaliana* and the three legumes examined. Chromosome numbers are indicated outside each figure as follows: in turquoise, *P. vulgaris*; in green, *L. japonicus*; in brown, *G. max*; and in purple, *A. thaliana*. The smaller synteny maps to the right of each image represent the synteny of all the genes in the genomes compared here.

Collinearity of genes was also examined between pairs of legume species that develop determinate nodules, namely *P. vulgaris*, *G. max*, and *L. japonicus*. The results indicate that 35 *CrRLK1L* genes have collinearity between *P. vulgaris* and *G. max*, 12 genes between *P. vulgaris* and *L. japonicus*, and 20 genes between *L. japonicus* and *G. max* (Figure 3A–C, Table S4). Collinearity between the *CrRLK1Ls* of these three legumes versus *A. thaliana* was also explored. This analysis revealed 24 legume genes that are syntenic to those of *A. thaliana*: 9 from *P. vulgaris*, 1 from *L. japonicus*, and 14 from *G. max (*Figure 3A–C, Table S4). We noted that in most of the syntenic gene pairs identified, the genes belong to the same clade. Thus, some *FER* genes in *P. vulgaris* are syntenic to *FER* genes in *L. japonicus* and *G. max*, and the same was true for some of the *MEDOS* and *HERK* genes *(*Figure 3A–C, Table S4). Moreover, some of the genes maintain this collinearity between legumes and *A. thaliana*. These data strongly suggest that these genes are orthologous.

### 3.5. Exon–Intron Structure of CrRLK1L Genes in Legumes and A. thaliana

**Figure 4:**
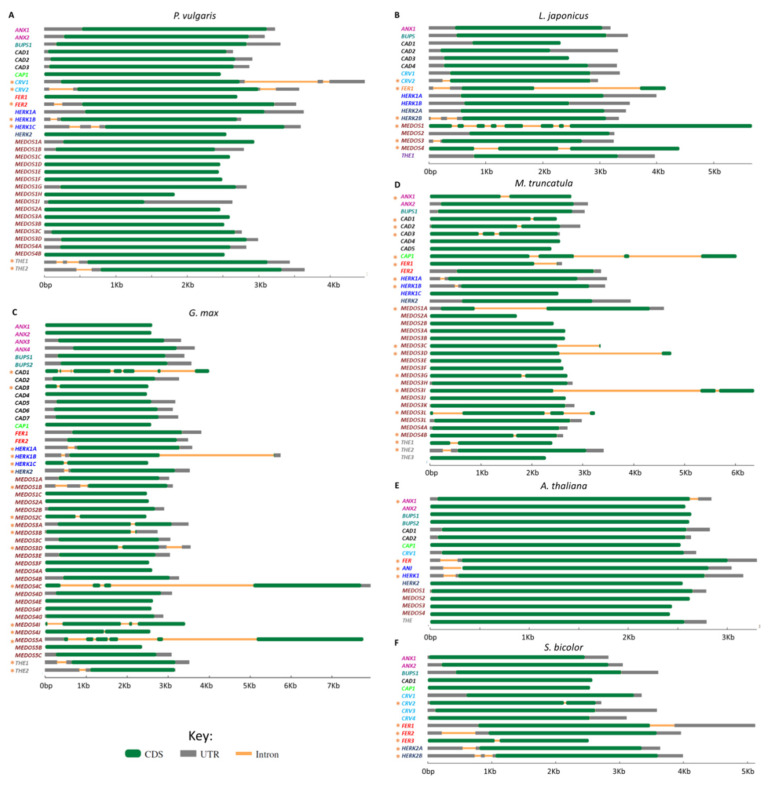
Gene structure of the *CrRLK1L* subfamily genes in four legumes, *A thaliana*, and *S. bicolor*. The exon–intron structures of all *CrRKL1L* genes from (**A**) *P. vulgaris*, (**B**) *L. japonicus*, (**C**) *G. max*, (**D**) *M. truncatula*, (**E**) *A. thaliana*, and (**F**) *S. bicolor* were analyzed using the Gene Structure Display Server database. Exons (CDS), introns, and untranslated regions (UTRs) are represented according to the key. Gene names are highlighted in colors as follows: *ANX* in purple, *BUPS* in green, *CAD* in black, *CAP* in lime, *CRV* in blue, *FER* in red, *HERK1* (*ANJ*) in blue, *HERK2* in dark blue, *MEDOS* in brown, and *THE* in gray. Orange asterisks to the left of the names indicate genes with introns.

To analyze the structural organization and evolution of the *CrRLKL1L* genes following their duplication, we analyzed the exon–intron distribution in these genes in *P. vulgaris*, *M. truncatula*, *G. max*, *L japonicus*, and *A. thaliana*, using Gene Structure Display Server 2.0 software for better visualization (Figure 4). Most of the *CrRLKL1L* genes in *P. vulgaris* have no introns (26 genes), though four of them have one intron (*PvCRV2*, *PvFER2*, *PvHERK1C*, and *PvTHE1*) and three have two introns (*PvCRV1*, *PvHERK1B*, and *PvTHE2*) (Figure 4A). In *L. japonicus*, half of the *CrRLK1L* genes have no introns, two have one intron (*LjCRV2* and *LjMEDOS3*), three have two introns (*LjFER*, *LjHERK2B*, and *LjMEDOS4*), and one has seven introns (*LjMEDOS1*) (Figure 4B). More than half of the *G. max CrRLK1L* genes have no introns, and 17 genes have one to seven introns (*GmHERK1A*, *GmHERK1B*, *GmHERK1C*, *GmHERK2*, *GmMEDOS1B*, *GmMEDOS2C*, *GmMEDOS3A*, *GmMEDOS3B*, *GmMEDOS3D*, *GmMEDOS4C*, *GmMEDOS4I*, *GmMEDOS4J*, *GmMEDOS5A*, *GmTHE1*, *GmTHE2*, *GmCAD1*, and *GmCAD3*) (Figure 4C). In *M. truncatula*, more than half of the genes (19 genes) have no introns, and 16 of them possess one to three introns (*MtANX1*, *MtCAP*, *MtFER1*, *MtHERK1A*, *MtHERK1B*, *MtMEDOS1A*, *MtMEDOS3C*, *MtMEDOS3D*, *MtMEDOS3G*, *MtMEDOS3I*, *MtMEDOS3L*, *MtMEDOS4B*, *MtTHE1*, *MtTHE2*, *MtCAD1*, *MtCAD2*, and *MtCAD3*) (Figure 4D). Most of the 17 *A. thaliana* genes have no introns, with only four of them having one intron (*AtANX1*, *AtANX2*, *AtFER*, and *AtHERK1*) (Figure 4E). Although some intron conservation was detected among the five plants analyzed, in *FER*, *HERK*, and *MEDOS* genes, many of the legume *CrRLK1L* genes have more introns than the corresponding *A. thaliana* genes.

### 3.6. Analysis of the Expression Patterns of the CrRLK1L Genes in Legumes, A. thaliana, and P. patens

**Figure 5:**
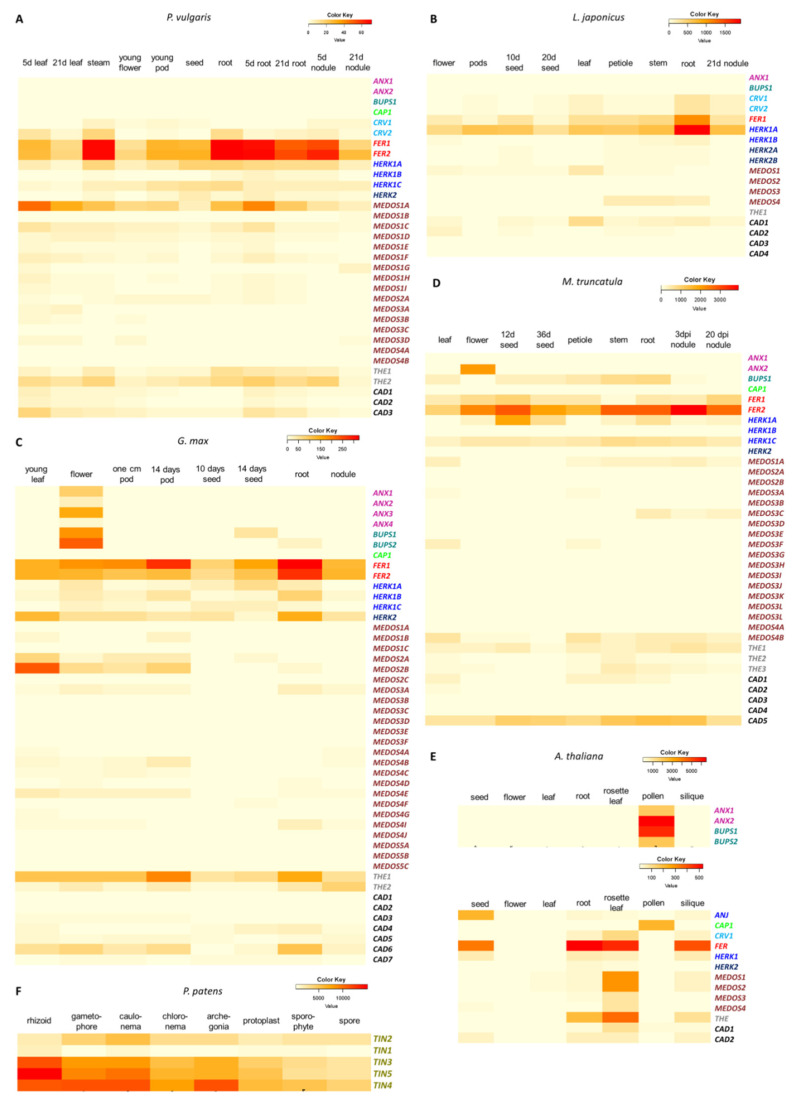
Gene expression profiles of *CrRLK1Ls* in four legumes, *A. thaliana*, and *P. patens*. Heat map of expression profiles of *CrRLK1L* in (**A**) *P. vulgaris*, (**B**) *L. japonicus*, (**C**) *G. max*, (**D**) *M. truncatula*, (**E**) *A. thaliana*, and (**F**) *P. patens*. Transcriptome data were extracted from the PvGEA, LotusBASE, and BAR databases. RPKM values are represented as color key codes above each heat map. Gene names are indicated as in the following color key; *ANX* in purple, *BUPS* in green, *CAP* in lime, *CRV* in blue, *FER* in red, *HERK1* (*ANJ*) in blue, *HERK2* in dark blue, *MEDOS* in brown, *THE* in gray, and *CAD* in black.

To evaluate the expression patterns of the *CrRLK1Ls* genes between different organs in four legumes and compare them with those in *A. thaliana* and *P. patens*, we retrieved and compared the expression data for *P. vulgaris CrRLK1Ls* from the common bean gene expression atlas (PvGEA) [60], for *L. japonicus* from the Lotus Base [49], for *M. truncatula* from MtGEA [61,62], and for *G. max*, *A. thaliana*, and *P. patens* from the BAR resource [61,63,64,65]. Expression data are represented as heat maps for each species (Figure 5).

As described in the following sections, it was observed that most of the genes showed similar expression patterns in the four legumes examined (*P. vulgaris*, *L japonicus*, *G. max*, and *M. truncatula)* and in *A. thaliana (FER*, *ANX*, *BUPS*, *CAP*, *HERK*, *THE*, *MEDOS*, and *CRV)*. Moreover, two genes differed in their expression patterns two to ten-fold in some tissues of the four legumes compared to their expression in *A. thaliana* (*CAD* and *MEDOS*). In addition, some *CrRLK1L* genes are expressed in legume nodules. Every *P. patens CrRLK1L* gene is expressed in all tissues analyzed; however, the levels of accumulation varied among the different tissues.

#### 3.6.1. *FER* Genes Are Broadly Expressed in All Tissues in Four Different Plant Species

*FER* is the most studied gene of the *CrRLK1L* subfamily in *A. thaliana*, and it has key roles in diverse plant processes [3,4,5,6,7,8,9,10,11,15,16,17,18,19,20,21,22,23,24,25,26,27,28,29,68,69,70]. *A. thaliana* has only one *FER* gene, which is expressed in almost every tissue, with transcript levels being especially high in roots and rosette leaves (Figure 5E). The *FER* genes in *P. vulgaris*, *L. japonicus*, and *G. max* are expressed at high levels in almost every tissue, similar to the expression pattern of *AtFER* in *A. thaliana*. The two *FER* genes identified in *P. vulgaris* (*PvFER1* and *PvFER2*) are mainly expressed in roots and stems (Figure 5A). The single *FER* gene in *L. japonicus* (*LjFER*) shows expression in all tissues analyzed, with the highest levels in roots and stems (Figure 5B). *G. max* has two *FER* genes (*GmFER1* and *GmFER2*), which are both expressed at high levels, mainly in roots and pods (Figure 5C). In *M. truncatula*, there are two *FER* genes, both widely expressed in the tissues analyzed. *MtFER1* is mainly expressed in nodules, and seeds, while *MtFER2* shows the highest expression levels in in nodule, root, and stem (Figure 5D). These expression patterns suggest a presumed conservation of *FER* gene function between *A. thaliana* and the legumes.

#### 3.6.2. *ANX*, *BUPS*, and *CAP* Genes Are Expressed Only in *A. thaliana* Pollen Tubes and *G. max* Flowers

Five *CrRLK1L* genes in *A. thaliana* have been reported to be essential for pollen tube growth, *AtANX1*, *AtANX2*, *AtBUPS1*, *AtBUPS2*, and *AtCAP* [30,31,32,36]. The expression of these five genes in *A. thaliana* is limited to pollen tubes and shows the highest accumulation levels of all *CrRLK1L* genes (Figure 5E). Four of these genes (*PvANX1*, *PvANX2*, *PvBUPS*, and *PvCAP)* are present in *P. vulgaris*, but no expression was detected according to PvGEA (Figure 5A). In *L. japonicus*, there are only two of these genes, *LjANX* and *LjCAP*, and no expression was detected in any of the tissues evaluated (Figure 5B). By contrast, in *G. max*, there are four *GmANX* and two *GmBUPS* genes, all of which exhibit expression in flower (Figure 5C); *GmCAP* show no expression in any of the tissues analyzed (Figure 5C). *M. truncatula* have two *ANX*, one *CAP*, and one *BUPS* gene. *MtANX1* and *MtCAP1* show no expression in any tissue examined, *MtANX2* are expressed exclusively in flowers, and *MtBUPS1* is expressed in several tissues (Figure 5D). Since the PvGEA and Lotus Base databases do not include expression data for pollen tubes, the expression pattern observed for *ANX*, *BUPS*, and *CAP* genes in *P. vulgaris* and *L. japonicum* probably resembles that of *A. thaliana*, while in *G. max* and *M. truncatula ANX* and *BUPS* genes probably expanded their expression to other tissues beyond pollen.

#### 3.6.3. *HERK* and *THE* Genes Are Expressed in Roots, Leaves, and Pods/Siliques

*HERK* and *THE* genes have been reported to regulate cell wall homeostasis in *A. thaliana* root and leaf, and *HERK* genes have also been reported to be essential for fertilization [4,33]. *AtHERK1*, *AtHERK2*, and *AtTHE1* show highest levels of expression in rosette leaves, roots, and siliques (Figure 5E). In common bean, *PvHERK1A* and *PvHERK1C* are expressed in almost every tissue analyzed, mainly in roots and pods (Figure 5A), *PvHERK1B* and *PvHERK2* show low expression, and *PvTHE1* and *PvTHE2* have the highest levels of expression in leaves, roots, and stems (Figure 5A). In *L. japonicus*, the four *HERK* genes show their highest expression in roots and nodules, followed by stem, leaves, and pods; *LjTHE* is not expressed (Figure 5B). In *G. max*, *GmHERK1A* and *GmHERK1C* are most strongly expressed in seeds, *GmHERK1B* in roots, and *GmHERK2* shows the maximum expression in roots and leaves, and the two *GmTHE* genes are primarily expressed in pods and roots (Figure 5C). In *M. truncatula*, meanwhile *MtHERK1A*, *MtHERK1C*, and *MtTHE1* are expressed in almost every tissues analyzed, mainly in roots and seeds, *MtHERK1B* and *MtHERK2* are not expressed at all (Figure 5D). These data indicate that *HERK* and *THE* genes, which are expressed mostly in roots, leaves, and siliques in *A. thaliana*, have similar expression patterns in legumes, although the latter have many more copies of these genes.

#### 3.6.4. *MEDOS* Genes Are Mostly Expressed in Leaves

It has recently been reported that *MEDOS* genes are important for regulating growth in the presence of metal ions in *A. thaliana* [38]. The four *AtMEDOS* genes are expressed mainly in rosette leaves (Figure 5E). In common bean, we detected 15 *PvMEDOS* genes. *PvMEDOS1A* is the third most expressed gene of all the *CrRLK1L* genes in this legume. *PvMEDOS1B*, *PvMEDOS3C*, *PvMEDOS4A*, and *PvMEDOS4B* show no expression in the analyzed tissues, and the other 10 *PvMEDOS* genes are expressed at low levels (Figure 5A). Despite these differences in transcript levels, *PvMEDOS* genes are mainly expressed in leaves and roots (Figure 5A). *L. japonicus* has four *LjMEDOS* genes. *LjMEDOS1* is mainly expressed in leaves, *LjMEDOS4* is mainly expressed in stem and petiole, and *LjMEDOS2-3* genes show low expression in all tissues tested (Figure 5B). In *G. max*, there are 24 *GmMEDOS* genes, most of which show low or no expression in the tissues evaluated (14 of 24 genes). Nine *GmMEDOS* genes are mostly expressed in leaves and pods, whereas *GmMEDOS4I* is most strongly expressed in roots (Figure 5C). Eighteen *MEDOS* genes were founded in *M. truncatula*, of them only five (*MtMEDOS1A*, *MtMEDOS3A*, *MtMEDOS3C*, *MtMEDOS3F*, and *MtMEDOS4B*) show low expression levels, mainly in leaves, petioles, and roots (Figure 5D). These data suggest some conservation in the expression of *MEDOS* genes in leaves of legumes and *A. thaliana*. Nonetheless, some of these genes probably have additional functions, in legumes, which could be related to their expression in other tissues (Figure 5).

#### 3.6.5. *CRV* Gene Expression Is Observed in Roots and Leaves, but Is Absent in *G. max*

CURVY (*CRV*) is a CrRLK1L receptor that is important for the development of leaves and seeds, as well as for the transition from vegetative to reproductive growth [37]. In *A. thaliana*, *AtCRV* is mainly expressed in rosette leaves, roots, and siliques (Figure 5E). In *P. vulgaris* there are two *CRV* genes, *PvCRV1* and *PvCRV2*, both of which are expressed at very low levels, mostly in stems, leaves, and roots (Figure 5A). *L. japonicus* also has two *LjCRV* genes, both with low expression, mainly in roots, leaves, and nodules (Figure 5B). No *CRV* genes were identified in the *G. max* and *M. truncatula* genomes, suggesting a possible loss of these genes during their evolution (Figure 5C,D). These observations indicate that expression of *CRV* genes in roots and leaves is conserved; however, the decrease in *CRV* expression in legumes and the loss of this gene in *G. max* and *M. truncatula* suggest a gradual loss of function.

#### 3.6.6. The Tissue Specificity of *CAD* Gene Expression Is Broader in Legumes Than in *A. thaliana*

The *A. thaliana AtCAD1* and *AtCAD2* genes, which have not yet been characterized, are mainly expressed in rosette leaves and roots (Figure 5E). The three *PvCAD* genes identified in common bean show low expression, mostly in leaves and inoculated roots (Figure 5A). *L. japonicus* has four *LjCAD* genes; *LjCAD1* exhibits high expression in roots, leaves, and nodules, whereas the other three show low expression (Figure 5B). Seven *GmCAD* genes were detected in *G. max*: *GmCAD1* and *GmCAD2* show low expression; *GmCAD3* and *GmCAD5* are mainly expressed in pods, leaves, and flowers; *GmCAD4* and *GmCAD7* are predominantly expressed in seeds and roots; and *GmCAD6* is expressed in roots, flowers, pods, and leaves (Figure 5C). Three of the five *CAD* genes in *M. truncatula* show no expression, *MtCAD1* is expressed at low levels in leaves, petiole and stems, and *MtCAD5* is expressed in most of the tissues, but mainly in stem, roots and nodules (Figure 5D). Thus, *CAD* genes are expressed in a wider range of tissues in legumes than in *A. thaliana*.

#### 3.6.7. *P. patens CrRLK1L* Genes Are Widely but Differentially Expressed in All Tissues Tested

Our phylogenetic analysis showed that the five *CrRLK1L* genes in *P. patens* (*PpTIN1-5*) are clustered in a single distinct clade (Figure 1, Figure S1). All five genes are abundantly expressed in every tissue analyzed (Figure 5F). *PpTIN1*, *PpTIN2*, and *PpTIN4* are mainly expressed in the rhizoid (an organ functionally related to the roots of land plants) and the caulonema (an organ necessary for colonization and nutrient acquisition). *PpTIN3* is mostly expressed in the archegonia (the female reproductive organs in the moss) and in the caulonema. Maximum levels of *PpTIN5* accumulation are observed in the caulonema and the gametophore (the tissue carrying the sex organs in moss) (Figure 5F). These variations in the expression patterns of the *P. patens CrRLK1L* genes suggest a certain amount of functional specialization of these genes in this moss, since the five genes probably originated from duplication of a single *CrRLK1L* gene.

#### 3.6.8. Certain *CrRLK1L* Genes Are Differentially Expressed during Nodulation

We observed that almost none of the legume *CrRLK1L* genes are expressed specifically in symbiotic organs; however, some of them are highly expressed in these symbiotic organs (Figure 5). In *P. vulgaris* there are at least nine of these genes (*PvCRV1*, *PvFER1*, *PvFER2*, *PvHERK1A*, *PvHERK1C*, *PvMEDOS1A*, *PvMEDOS1C*, *PvCAD3*, and *PvTHE2*) (Figure 5A), nine genes in *L. japonicus* (*LjCRV1*, *LjCRV2*, *LjFER1*, *LjHERK1A*, *LjHERK2A*, *LjHERK2B*, *LjMEDOS4A*, *LjCAD1*, and *LjCAD3*) (Figure 5B), 10 genes in *G. max* (*GmFER1*, *GmFER2*, *GmHERK1C*, *GmHERK2*, *GmMEDOS3A*, *GmMEDOS4*, *GmCAD1*, *GmCAD3*, *GmTHE1*, and *GmTHE2*) and nine genes in *M. truncatula* (*MtFER1*, *MtFER2*, *MtHERK1A*, *MtHERK1C*, *MtMED1A*, *MtMED3C*, *MtMEDOS4B*, *MtCAD5*, and *MtTHE1*) (Figure 5C–D). We noticed that several of the 37 genes expressed in nodules are shared among the four legumes (Figure S5). *FER1* is expressed in nodules in all four legumes, whereas five genes where shared between three different legumes: *CAD3* is shared between *P. vulgaris*, *L.* *japonicus*, and *G. max*; *HERK1A* is shared between *P. vulgaris*, *L.* *japonicus*, and *M. truncatula*; while *L.* *japonicus*, *G. max*, and *M. truncatula* share *MEDOS4;* and *FER2* and *HERK1C* are shared between *P. vulgaris, G. max*, and *M. truncatula*. Moreover, seven genes were founded in nodules in two legume pairs; *P. vulgaris* and *L. japonicus* nodules express *CRV1*, *THE2* is expressed in nodules of *P. vulgaris* and *G. max*, and *MEDOS1A* in *P. vulgaris* and *M. truncatula*. Furthermore, *HERK2A* and *CAD1* are expressed in *G. max* and *L. japonicus*, while *MEDOS3* and *THE1* are shared between *G. max* and *M. truncatula.* This comparative analysis also revealed four nodule-expressed genes that were exclusive to one legume (Figure S5).

These data together indicate that along with the highly conserved expression profiles of *CrRLK1L* genes in legumes, some of them are differentially expressed in nodules, suggesting a possible role of these genes in the nodulation process.

### 3.7. Expression of CrRLK1L Genes in P. vulgaris Nodules

To validate the expression profile of some *CrRLK1L* genes in nodules that we observed in the PvGEA data [60], as well as to describe their expression patterns during different stages of nodulation, we selected eight *P. vulgaris CrRLK1L* genes for further investigation: *PvFER1*, *PvFER2*, *PvHERK1A*, *PvHERK1C*, *PvMEDOS1A*, *PvMEDOS1C*, *PvTHE2*, and *PvCAD3.* Expression of these genes was measured at four stages of the *P. vulgaris*-*R. tropici* symbiosis: 5, 7, 14, and 21 days post-inoculation (dpi) of wild-type roots. The eight genes were differentially expressed at the different stages of nodulation, corroborating their presumed role during nodulation in common beans.

**Figure 6:**
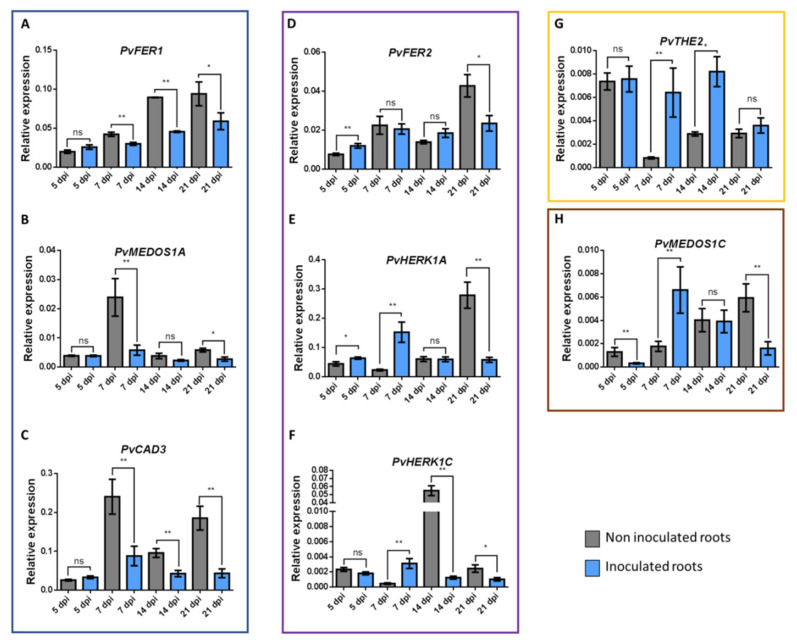
RT-qPCR expression analysis of eight *P. vulgaris CrRLK1L* genes. Relative expression profiles of eight *CrRLK1L* genes from *P. vulgaris* roots inoculated or not with *R. tropici*. Genes were classified into four groups according to their expression; the blue box indicates downregulated genes at the early and late time points: *PvFER1* (**A**), *PvMEDOS1A* (**B**), and *PvCAD3* (**C**); the purple box shows genes whose expression is upregulated at the early time points assessed, but downregulated later on: *PvFER2* (**D**), *PvHERK1A* (**E**), and *PvHERK1C* (**F**); the yellow box indicates the expression of *PvTHE2* (**G**), which was upregulated at the early and late time points; and the brown box displays *PvMEDOS1C* (**H**), showing variable expression at the different time points evaluated. The transcript accumulation of the selected genes was assessed by RT-qPCR and normalized according to *elongation factor 1α* (*ef1α*) gene expression. Blue bars represent inoculated roots, whereas gray bars indicate the expression levels in non-inoculated roots. The error bars represent standard deviation of the mean (*n* = 6). A Student’s *t*-test was performed to evaluate significant differences, * represents *p* ≤ 0.05, ** represent *p* ≤ 0.01, ns represents non-significant difference.

These eight genes displayed four different expression profiles. Three genes were suppressed in at least one of the nodulation steps analyzed (blue box, Figure 6). *PvFER1* and *PvCAD3* showed reduced transcript accumulation in inoculated roots at 7, 14, and 21 dpi compared to uninoculated roots, whereas no differences were observed at 5 dpi (Figure 6A,B). *PvMEDOS1A* was downregulated at 7 and 21 dpi in inoculated roots but was expressed at similar levels regardless of inoculation at 5 and 14 dpi (Figure 6C). Three genes were upregulated in the early stages (5 or 7 dpi) but then suppressed in the later stages (14 or 21 dpi) (purple box, Figure 6); *PvFER2* and *PvHERK1C* were upregulated in inoculated roots at 5 dpi, and *PvHERK1A* was upregulated at 5 and 7 dpi. At 21 dpi, however, *PvFER2*, *PvHERK1C*, and *PvHERK1A* were downregulated in inoculated roots compared to the controls, as was *PvHERK1C* at 14 dpi (Figure 6D–F). A third expression pattern was displayed by *PvTHE2*; transcripts of this gene showed increased accumulation in inoculated roots at 7 and 14 dpi relative to the controls but at 5 and 21 dpi, levels of transcript accumulation were similar to the controls (green box, Figure 6G). Finally, *PvMEDOS1C* showed fine-tuned changes in expression; relative to the controls, transcript accumulation for this gene was decreased at 5 and 21 dpi, increased at 7 dpi, and unchanged at 14 dpi (brown box, Figure 6H).

These data indicate that the eight genes analyzed here are indeed differentially expressed in common bean roots at different stages of the nodulation process and probably perform different functions throughout the symbiotic process.

## 4. Discussion

### 4.1. Structural Features of CrRLK1L Genes

The RLK subfamily CrRLK1L has emerged as an important signaling component of numerous biological processes, including development, immune responses, and fertilization, among others. Previous studies have analyzed the phylogeny of *CrRLK1L* genes in *A. thaliana*, rice (*O. sativa*), cotton (*Gossypium hirsutum*), and pear (*Pyrus bretschneideri*), as well as their expression under different conditions [68,69,70]. However, it is important to extend these studies to other agro-ecologically important crops, such as legumes, which have the ability to fix nitrogen in association with the soil bacteria rhizobia. Furthermore, a comprehensive phylogenetic study of the CrRLK1L subfamily in a larger number of plant species will yield new information about the functions of these proteins and their evolutionary paths since their appearance in the plant kingdom. The usefulness of this bioinformatic approach is evident in the current study, in which we were able to analyze the CrRLK1L subfamily in model legumes and common bean, using different “*in silico*” approaches, and thereby elucidate its possible functions in the legume-rhizobia mutualistic interaction.

We identified 1050 CrRLK1L proteins from the 57 embryophytes included in this analysis, which fell into 11 phylogenetically distinct clades (Figure 1A, Figure S1, Table S2). Chlorophytes lack this plant-specific RLK subfamily, indicating that it arose during the transition from chlorophytes to embryophytes, which probably occurred about 500 million years ago (mya) [71]. A feature that differentiates embryophytes from other plants is their sexual reproduction [71,72] and, since some CrRLK1Ls are key regulators of fertilization [4,16,30,31,33,36], this may link the emergence of the CrRLK1L subfamily with the advent of embryophytes. We found that bryophyte CrRLK1Ls cluster together in a unique clade (TINIA), whereas the land plant proteins are distributed among the remaining ten clades (Figure 1A, Figure S1, Table S2). The number of CrRLK1Ls in these mosses varies from one to seven, revealing the first duplication events of the *CrRLK1L* lineage. Monocots and eudicots diverged around 150 mya, and have evolved along different evolutionary paths [73]. The subsequent eudicot radiation, dated around 100 mya, has been associated with polyploidization events [74]. Interestingly, there are more CrRLK1L proteins in eudicots than in monocots, demonstrating the different evolutionary fates of the genes of this subfamily in monocots and eudicots. Some eudicots have particularly large numbers of these proteins compared to other eudicots (Figure 1A–B, Table S1). This increase in the number of CrRLK1Ls could be associated with the appearance of the MEDOS clade (present in all eudicots but in only a few monocots) and with subsequent expansion of the MEDOS clade in eudicots.

Our phylogenetic analysis of CrRLK1Ls from four legumes, *A. thaliana*, and *P. patens*, revealed 155 genes distributed in 11 clades (Figure S2A). As expected, the MEDOS clade had the most members. We observed that the proteins in this clade are clustered on one chromosome in *A. thaliana* and *L. japonicus*, while in *P. vulgaris*, *G. max*, and *M. truncatula*, these genes form two clusters (Figure S3). Some reports indicate that in plants the expansion of gene subfamilies mainly occurred through dispersed, tandem, and whole-genome duplications [75,76,77,78,79,80]. In pear, most *CrRLK1L* genes arose by whole-genome duplication and some by dispersed gene duplications [70]. The tandem duplications we observed suggest that, in the analyzed legumes and in plants with a high number of *CrRLK1L* genes, the *MEDOS* genes arose from tandem duplications, whereas the other *CrRLK1L* genes probably arose from whole-genome or segmental duplications.

The exon-intron structure of genes has been associated with gene function, and it affects RNA splicing, RNA stability, and chromatin organization [81,82,83]. Exon-intron patterns have been used to reveal time evolution, constant variation, and their co-variations [84]. Our comparative analysis of the exon–intron distribution in *CrRLK1L* genes revealed that, compared to *A. thaliana*, legumes have more *CrRLK1L* genes with introns and more introns in each gene (Figure 4). Nevertheless, the expression patterns of the legume *CrRLK1L* genes were similar to those of the corresponding orthologs in *A. thaliana* (Figure 5). In pear, it has been proposed that the *CrRLK1L* genes have lost introns, but their expression patterns are similar to those of *A. thaliana* and rice genes [85]. Our observations suggest that the increase in the number of introns is associated with duplication and evolutionary events, but these have little or no effect on gene function and expression.

Analysis of the synteny of the *CrRLK1L* genes revealed homology between some gene pairs in the plants analyzed. In *P. vulgaris*, *L. japonicus*, and *A. thaliana*, four, one, and two syntenic gene pairs were identified, respectively; each gene observed was syntenic with only one additional gene (Figure S4). By contrast, 21 syntenic gene pairs were identified in *G. max*, and some genes have synteny with more than one other gene (Figure S4). The higher number of syntenic genes in *G. max* is probably because of the polyploidization event that occurred in this legume [86]. Compared to the number of syntenic *CrRLK1L* genes we observed between *P. vulgaris* and *G. max*, there were fewer between either of these species and *L. japonicus*, and even less between *P. vulgaris* and *A. thaliana* (Figure 3). There is a clear correlation between the degree of synteny and the time of divergence between species [74,76]. The degree of synteny also depends on the evolution of the genome; in angiosperms, whole-genome duplication and subsequent gene loss have driven plant evolution and have also reduced collinearity across species [77,87]. Our data are consistent with an early divergence between *P. vulgaris* and *G. max*, compared to *L. japonicus*, and an even longer divergence time between *P. vulgaris* and *A. thaliana*.

The characteristics of a protein are important for its activity and correspond with taxonomy, environmental adaptation, subcellular localization, and genome size [85,88]. From this perspective, the contrast between the characteristics of CrRLK1Ls from legumes versus *A. thaliana* denotes greater variability in the legume sequences and correlates with larger genomes (Table 2). A protein’s iP reflects its amino acid composition and conformation and determines its activity [89]; the wider iP ranges and longer sequences of the legume CrRLK1Ls could reflect specialization of some of these proteins for different tissues or processes, possibly giving these plants better adaptability to environmental changes. In the five plant species studied here, we observed a high conservation of overrepresented motifs in all of the CrRLK1Ls (Figure 2). The conservation of these motifs, which are located in the malectin and kinase domains characteristic of this subfamily, indicates their importance for protein activity.

### 4.2. Differences and Similarities in the Expression of CrRLK1L Genes in Legumes and in A. thaliana

Previous studies in *A. thaliana* have reported that *CrRLK1L* genes participate in a variety of processes, such as development, cell communication, and plant-microbe interactions (Table 1), and that the functions of these genes correspond with their expression profiles (Figure 5E). A previous study comparing *CrRLK1L* gene expression in pear and *A. thaliana* reported that the expression profiles of some genes are conserved between these species; however, the expression of many other genes was lost or altered in pear compared to *A. thaliana* [70]. We performed a comparative in silico analysis of *CrRLK1L* gene expression profiles in four legumes and *A. thaliana* and observed that the expression patterns of most of the genes are conserved. *FER*, *HERK*, *THE*, *CRV*, and *MEDOS* showed similar expression profiles in the five species examined; these genes are expressed in almost all tissues. In *A. thaliana*, *ANX*, *BUPS*, and *CAP* are pollen-specific genes. These genes are not expressed at detectable levels in *P. vulgaris* or *L. japonicus*, at least in the tissues included in the databases (Figure 5). However, since there is no data available for expression of these genes in pollen or pollen tubes, we propose that these genes could also be pollen-specific in these legumes, as they are in *A. thaliana*. The expression profiles of some of the legume *CAD* genes differed by two to ten-fold from those of the *AtCAD* genes in some tissues, suggesting additional functions for these genes in legumes.

Legumes are characterized by the ability to form nodules that house endosymbiotic rhizobia. This relationship generates a driving force between the two symbionts that leads them to co-evolve [90,91]. It has been reported that plant lipochitooligosaccharide receptors acquired symbiotic functions before gene duplication [92]. In the four legumes analyzed here, some *CrRLK1L* genes showed transcript accumulation in nodules, suggesting that these genes have been recruited to the symbiotic process, in addition to any other roles they may have. We identified nine genes that were expressed in nodules in *P. vulgaris*, nine in *L. japonicus*, ten in *G. max*, and nine in *M. truncatula*. Among those genes, *FER1* is expressed in nodules of all four legumes, (Figure 5, Figure S5), five other nodule-expressed genes were shared between three of the four legumes, seven shared by different pairs of legumes, and four genes were expressed in nodules of only one of the legumes (Figure 5, Figure S5). These data may suggest that some *CrRLK1L* genes participate in the symbiotic process. Nonetheless, further functional analyses are needed to test this hypothesis.

### 4.3. Putative Roles of CrRLK1L Genes during Nodulation

**Figure 7:**
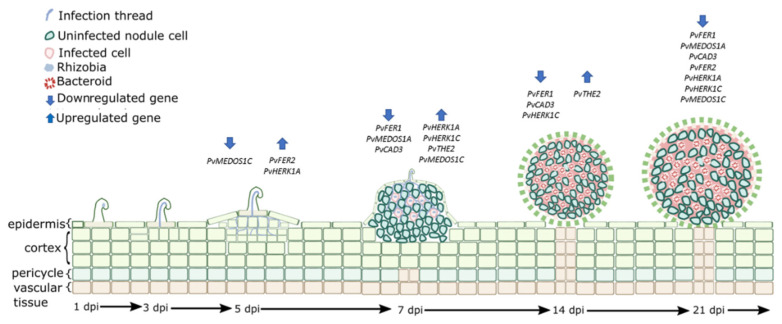
Putative roles of the eight *CrRLK1L* genes evaluated during nodule organogenesis in common bean. Graphic representation of the proposed roles of the eight *CrRLK1L* genes of common bean, based on the RT-qPCR data obtained in this study. Different stages corresponding to days post-inoculation (dpi) are observed. At the early stages, the bacteria and the root establish a molecular dialogue, which promotes curling of the root hair where the bacteria are enclosed in an infection chamber (not shown). One to three days before the bacteria become trapped, an infection thread (IT) is formed, which advances through the infected root hair cell, reaching the outer cortex of the root. Concurrently, cortex cells de-differentiate and divide. By 5 dpi, dividing cells in the outer cortex generate a nodule primordium, whereas the IT branches toward the primordium. By 1 nitrogen fixation rates.

We examined the expression profiles of eight *CrRLK1L* genes in *P. vulgaris* roots inoculated with *R. tropici* and found that these genes were differentially expressed at different stages of nodule development. Figure 7 provides a schematic summary of the nodulation process in *P. vulgaris* and the steps in which the eight genes presumably participate. At 5 dpi, the nodule primordia begin to emerge from the root epidermis, and the infection thread, filled with bacteria, penetrates the outer cortex of the root and branches [93,94,95,96,97]; at this point, *PvMEDOS1C* was downregulated in the inoculated roots relative to the control, whereas *PvFER2* and *PvHERK1A* were upregulated (Figure 7). By 7 dpi, many nodules have already emerged from the root epidermis, and some nodule primordia cells contain bacteria that have been released from the infection threads [95,96,97,98,99]; at this time, four genes (*PvHERK1A*, *PvHERK1C*, *PvTHE2*, and *PvMEDOS1C*) showed high expression, whereas three others (*PvFER1*, *PvMEDOS1A*, and *PvCAD3*) exhibited low expression in inoculated roots relative to the control (Figure 7). Some *CrRLK1Ls* have been reported to be important regulators of cell expansion, cell wall maintenance, and membrane integrity during cell growth [3,32,100,101,102]. The *P. vulgaris CrRLK1L* genes that are induced at 5 and 7 dpi could be supporting similar functions, since at these nodulation stages, there are high rates of cell division and expansion [97,103,104,105,106,107]. Likewise, internalization of the bacteria depends on growth and branching of the infection thread through the root cortex and subsequent release of the bacteria into the cells of the nodule primordia [97,98,99]. Some *CrRLK1L* genes have been reported to be regulators of immune responses [9,11,12,20,108], indicating that downregulation of some *CrRLK1L* genes at this stage of nodulation might inhibit pathogen responses during infection.

At 14 dpi, the bacteria within the infected cells differentiate into bacteroids and most of the nodules are matured, initiating nitrogen fixation [93,95,96,109,110,111]. At this stage, three genes, *PvFER1*, *PvCAD3*, and *PvHERK1C*, were downregulated and *PvTHE2* was upregulated in inoculated roots relative to non-inoculated ones (Figure 7). The downregulated genes could be associated with avoidance of immune responses, as earlier in the nodulation process. In addition, the downregulated genes could be involved in regulating nitrogen flow, considering that, in *A. thaliana*, FER has been reported to be a growth regulator that responds to the C/N ratio [14]. Downregulation of some *CrRLK1Ls* may be necessary to promote nitrogen fixation. The upregulation of *PvTHE2* suggests that this gene may be associated with other functions during this stage, such as nodule development. At 21 dpi, common bean nodules are fully developed and display high rates of nitrogen fixation [93,96,110,111]. *PvFER1*, *PvFER2*, *PvHERK1A*, *PvHERK1C*, *PvMEDOS1A*, *PvMEDOS1C*, and *PvCAD3* were downregulated at 21 dpi (Figure 7). The downregulation of most of the *CrRLK1L* genes could be related to the end of nodule development and to deactivation of immune responses to maintain symbiosis and nitrogen fixation at the highest levels.

ROS are important signaling molecules that participate in nodule organogenesis processes associated with CrRLK1Ls. In plant cells, ROS are mainly produced through the activity of respiratory burst oxidase homologs (RBOHs in plants), which are called NADPH oxidases in mammals [112]. RBOH-dependent ROS production has been described as a conserved mechanism in CrRLK1L activity; FER, ANX1, and ANX2 promote phosphorylation, and thereby activation, of RBOH, inducing ROS-mediated polar growth in pollen tubes and root hairs in *A. thaliana* [10,32]. In *P. vulgaris* and *M. truncatula*, ROS signaling is essential for initiating root hair cell responses to the presence of rhizobia. Impairment of ROS production through downregulation of *Rbohs* in these species inhibits the progression of infection thread growth in *P. vulgaris* (*PvRbohA* and *PvRbohB)* and swelling of root hair tips in *M. truncatula* (*MtRbohB* and *MtRbohE*) [113,114,115]. In addition, previous studies have revealed *Rboh* promoter activity associated with cell division in the cortex and vascular bundles of nodules, suggesting a possible role of these oxidases in nodule development [114,115,116,117]. Similarly, *Rboh* genes are differentially expressed during nodulation in *P. vulgaris*, *L. japonicus*, and *M. truncatula* [114,115,117,118], as we observed for eight nodule-expressed *CrRLK1L* genes in common bean. Altogether, these data suggest that the *CrRLK1L* genes may be participating in the nodulation process through regulation of ROS signaling at specific stages of nodule organogenesis.

Phytohormones also appear to have roles in nodulation. For instance, studies in several legumes have reported that abscisic acid (ABA) is a negative regulator of nodulation [119,120] but that it also has some positive effects on the growth and functioning of nodules [121,122]. In pea (*Pisum sativum*) and soybean (*G. max*), brassinosteroid (BR) inhibits nodulation in some studies [123,124], whereas in peanut (*Arachis hypogaea*) and *P. vulgaris*, some studies show positive effects of BR on nodulation [125,126]. Jasmonic acid (JA) has both positive and negative effects on nodulation, depending on the legume species and the stage of nodule development at which it is applied [119,127,128,129]. Ethylene has mainly been associated with negative regulation of nodulation [130,131]. RALF peptide hormones have been reported to be negative regulators of infection and nodule organogenesis in *M. truncatula* [45]. Some CrRLK1Ls are known to be RALF receptors [8,39,40,101]. In *A. thaliana*, the expression of *FER*, *THE*, and *HERK* is induced by BRs [4] and FER is a hormone response modulator, fine-tuning ethylene and BR signaling during hypocotyl growth [5] and suppressing ABA and JA signaling [6,9]. In the current study, we found that two *FER* genes, two *HER* genes, and one *THE* gene were differentially expressed at different stages of nodulation in common bean. These results, along with the previously described roles for these genes in regulating hormone signaling, allow us to speculate that these genes may participate in nodulation through the regulation of hormone signaling at several stages of nodule organogenesis. Nonetheless, experimental evidence is needed to test this hypothesis.

In this work, we examined eight differentially expressed *CrRLK1L* genes at different stages of nodulation in common bean. Based on our results, we postulate that these proteins are regulators in this process. Forthcoming reverse genetics experiments in common bean will expand our knowledge of the particular roles of these *CrRLK1L* genes in nodulation. Our analysis of previously published transcriptomic data [60,61,63,64] demonstrated that related *CrRLK1L* genes are expressed in nodules of other legumes. Based on the phylogenetic, syntenic, and expression profiling analyses reported here, we predict that *CrRLK1L* subfamily homologs in other legumes may have a conserved role in nodulation, as these genes presumably do in common bean.

## 5. Conclusions

In this study, we identified 1050 CrRLK1L proteins in 57 plant species, clustered into 11 clades, one of them specific to moss and clubmoss proteins. This receptor subfamily probably appeared with the emergence of land plants, since no homologous proteins were detected in chlorophytes. In silico analysis in legumes and *A. thaliana* revealed that these receptors have expanded mostly by whole-genome and isolated duplication, and in the case of the *MEDOS* clade, by tandem duplication. Moreover, this analysis revealed high conservation of gene and protein structure and high similarities in expression profiles, suggesting analogous functions. Remarkably, RT-qPCR quantification of transcript levels in *P. vulgaris* roots inoculated with *R. tropici* revealed that some *CrRLK1L* genes could have different roles at different stages of the nodulation process. Considering the genomic similarities observed, we speculate that these roles in nodule organogenesis could be conserved in other legumes.

## Figures and Tables

**Table 1:** *CrRLK1L* genes studied in different plant species.

Gene Name	Plant Species	Mutant/RNAi	Phenotype	Reference
*FERONIA*	*A. thaliana*	*fer*	PT overgrowth, multiple PT reach one ovules	[15,16,17,18,19]
		*fer*	Collapsed, burst and short RH	[10]
		*fer*	Resistance to Powdery mildew infection, increased susceptibility to *Pseudomonas syringae* pv. *tomaeum* DC3000	[11,20]
		*fer*	Ethylene hypersensitivity, brassinosteroid insensitivity, abscisic acid hypersensitivity, an increase of s-adenosyl methionine synthesis, inhibition of jasmonic acid responses	[5,6,7,9]
		FER RNAi	Dwarf phenotype	[4,21]
		*fer*	Larger seed size	[22]
		*fer*	Salt hypersensitivity	[23]
		*fer*	Increased starch accumulation in a sucrose medium. Hypersensitivity to high carbon/nitrogen ratio	[14,24]
		*fer*	Reduced induction of ErbB3-binding protein 1, alteration of ribosome synthesis	[13,25]
		*fer*	Delay in the flowering time under long day condition	[26]
		*fer*	Hypersensitivity to nickel, tolerance to cadmium, coper, zinc, and lead	[27]
	*Oriza sativa* L.	*flr2*	Enhanced resistance to *Magnaporthe oryzae* infection	[28]
		*flr11*	Enhanced resistance to *M. oryzae* infection	[28]
	*Fragaria x ananassa*	FaMRL47RNAi	Fruit ripening acceleration	[29]
*BUPS1/2*	*A. thaliana*	*bups1*	PT overgrowth	[30]
		*bups1 bups2*	Enhanced bups1 phenotype: PT overgrowth	
*ANXUR1/2*	*A. thaliana*	*anx1 anx2*	Reduced fertility, PT burst	[19,31,32]
*HERKULES1*	*A. thaliana*	*herk1 the*	Dwarf plants	[19,27,33]
		*herk1*	Tolerance to cadmium, coper, nickel, and zinc	
		*herk1*	PT overgrowth	
*HERKULES2*	*A. thaliana*	*herk2*	Tolerance to cadmium, coper, nickel, and lead	[27]
*ANJEA*	*A. thaliana*	*herk anj*	PT overgrowth	[33]
*THESEUS1*	*A. thaliana*	*the prc*	Rescues of hypocotyl growth but without *prc* cellulose deficiency phenotype	[34]
		*the*	Hypersensitivity to lead and zinc, tolerance to nickel	[27]
		*herk1 the*	Dwarf plants	[4]
*CAP*	*A. thaliana*	*cap*	Altered PT growth in low calcium	[35]
		*cap*	RH bursting and bulging	[36]
*CURVY*	*A. thaliana*	*crv*	Distortion of trichomes, altered pavement morphology	[37]
*MEDOS1-4*	*A. thaliana*	*med1,2,3,4*	Reduced growth in presence of metal ions	[38]

PT: pollen tubes, RH: root hairs, *prc: A. thaliana* mutant *procuste.*

**Table 2:** Transcript lengths and protein properties of the *CrRLK1L* subfamily members in *P. vulgaris*, *G. max*, *A. thaliana*, *L. japonicus*, and *M. truncatula*.

Gene ID *	Gene Name	CDS Length, bp	Protein Length, aa	iP	Molecular Weight, kDa
***P. vulgaris***					
Phvul.006G102700	*ANX1*	2589	862	5.71	95.82
Phvul.007G188300	*ANX2*	2589	862	5.6	96
Phvul.011G210400	*BUPS*	2670	889	5.41	97.19
Phvul.003G188000	*CAP*	2469	822	5.91	92.06
Phvul.004G109500	*CRV1*	2505	834	5.49	92.38
Phvul.007G074000	*CRV2*	2535	844	5.7	93.64
Phvul.008G081000	*FER1*	2700	899	5.99	98.37
Phvul.008G082400	*FER2*	2697	898	6.67	98.1
Phvul.005G139800	*HERK1A*	2514	837	5.64	92.51
Phvul.008G000200	*HERK1B*	2472	823	5.38	92.18
Phvul.011G069600	*HERK1C*	2514	837	5.79	92.94
Phvul.006G127900	*HERK2*	2547	848	7.3	93.01
Phvul.004G038800	*MEDOS1A*	2676	891	7.29	99.43
Phvul.004G039200	*MEDOS1B*	2235	744	7.05	83.82
Phvul.004G039600	*MEDOS1C*	2598	865	5.78	96.98
Phvul.004G039700	*MEDOS1D*	2406	801	5.79	90.35
Phvul.004G039800	*MEDOS1E*	2442	813	5.99	92.04
Phvul.004G039900	*MEDOS1F*	2490	829	6.51	93.64
Phvul.004G040000	*MEDOS1G*	2460	819	7.31	92.32
Phvul.004G040300	*MEDOS1H*	1824	607	6.26	68.13
Phvul.004G040901	*MEDOS1I*	1353	450	8.46	50.59
Phvul.004G039400	*MEDOS2A*	2463	820	5.99	92.31
Phvul.008G030200	*MEDOS3A*	2595	864	6.15	96.81
Phvul.008G030400	*MEDOS3B*	2517	838	6.24	93.86
Phvul.008G030700	*MEDOS3C*	2577	858	6.28	96.37
Phvul.008G030800	*MEDOS3D*	2601	866	4.83	97.08
Phvul.003G038700	*MEDOS4A*	2385	794	8.67	89.03
Phvul.003G038800	*MEDOS4B*	2523	840	5.95	93.94
Phvul.005G085600	*THE1*	2523	840	5.93	92.73
Phvul.011G148700	*THE2*	2538	845	5.87	93.05
Phvul.003G239300	*CAD1*	2499	832	6.63	93.06
Phvul.003G239400	*CAD2*	2589	862	8.33	96.18
Phvul.003G239500	*CAD3*	2586	861	5.86	96.32
***G. max***					
Glyma.03G247800	*ANXUR1*	2610	869	5.24	96.22
Glyma.10G163200	*ANXUR2*	2589	862	5.67	95.95
Glyma.19G245800	*ANXUR3*	2601	866	5.31	95.71
Glyma.20G225800	*ANXUR4*	2532	843	5.8	93.73
Glyma.12G235900	*BUPS1*	2637	878	5.77	96.33
Glyma.13G201400	*BUPS2*	2610	869	5.85	95.29
Glyma.17G102600	*CAP1*	2586	861	6.55	95.69
Glyma.09G273300	*FERONIA1*	2691	896	5.64	98.07
Glyma.18G215800	*FERONIA2*	2685	894	5.66	97.76
Glyma.12G074600	*HERKULES1A*	2514	837	5.86	92.74
Glyma.15G042900	*HERKULES1B*	2226	741	7.93	81.93
Glyma.U033500	*HERKULES1C*	2436	811	6.5	89.91
Glyma.09G024700	*HERKULES2*	2559	852	5.59	93.59
Glyma.02G121900	*MEDOS1A*	2463	820	8.23	92.15
Glyma.02G122000	*MEDOS1B*	1944	647	5.81	72.73
Glyma.02G196000	*MEDOS1C*	2481	826	5.83	93.46
Glyma.08G248900	*MEDOS2A*	2529	842	6.25	92.88
Glyma.08G249200	*MEDOS2B*	2616	871	6.24	96.62
Glyma.08G249400	*MEDOS2C*	2373	790	6.02	88.62
Glyma.13G054400	*MEDOS3A*	2691	896	6.11	99.53
Glyma.13G053800	*MEDOS3B*	2109	702	6.44	77.97
Glyma.13G053700	*MEDOS3C*	2460	819	5.63	91.47
Glyma.13G053600	*MEDOS3D*	2685	894	5.9	99.35
Glyma.13G054200	*MEDOS3E*	2364	787	8.48	88.74
Glyma.13G054300	*MEDOS3F*	2535	844	5.98	94.104
Glyma.18G269900	*MEDOS4A*	2610	869	6.26	97.24
Glyma.18G270100	*MEDOS4B*	2607	868	6.09	97.39
Glyma.18G270600	*MEDOS4C*	3372	1123	5.77	124.54
Glyma.18G270700	*MEDOS4D*	2574	857	6.25	95.77
Glyma.18G270900	*MEDOS4E*	2628	875	5.82	97.45
Glyma.18G271000	*MEDOS4F*	2592	863	5.9	96.86
Glyma.18G271100	*MEDOS4G*	2652	883	6.02	98.05
Glyma.18G270800	*MEDOS4I*	2730	909	5.98	102.67
Glyma.18G271200	*MEDOS4J*	2550	849	5.83	95.14
Glyma.19G033100	*MEDOS5A*	3561	1186	6.49	133.95
Glyma.U027000	*MEDOS5B*	2364	787	8.48	88.76
Glyma.U027100	*MEDOS5C*	2460	819	5.75	91.75
Glyma.12G148200	*THESEUS1*	2541	846	5.68	93.19
Glyma.12G220400	*THESEUS2*	2070	689	6.44	75.72
Glyma.05G099900	*CAD1*	2382	793	6.3	88.42
Glyma.05G100000	*CAD2*	2517	838	5.58	94.02
Glyma.09G133000	*CAD3*	2457	818	7.04	99.92
Glyma.10G231500	*CAD4*	2481	826	7.94	91.86
Glyma.16G179600	*CAD5*	2322	773	8.81	86.06
Glyma.17G166200	*CAD6*	2523	840	5.8	93.93
Glyma.20G162300	*CAD7*	2523	840	8.17	93.19
***A. thaliana***					
AT3G04690	*ANX1*	2837	895	6.47	98.16
AT5G28680	*ANX2*	2577	850	6.54	94.06
AT4G39110	*BUPS1*	2637	858	5.76	94.31
AT2G21480	*BUPS2*	2616	873	5.66	97.18
AT5G61350	*CAP*	2529	880	5.92	97.96
AT2G39360	*CRV*	2683	815	6.13	91.33
AT3G51550	*FER*	3298	830	5.82	91.48
AT5G59700	*HERK/ANJ*	3041	849	5.76	93.96
AT3G46290	*HERK1*	3158	855	5.91	93.31
AT1G30570	*HERK2*	2550	842	6.16	92.7
AT5G39000	*MEDO2*	2622	878	5.75	96.52
AT5G38990	*MEDOS1*	2785	871	5.51	95.95
AT5G39020	*MEDOS3*	2442	829	6.5	91.97
AT5G39030	*MEDOS4*	2421	824	5.65	91.84
AT5G54380	*THE1*	2789	834	5.7	93.39
AT5G24010	*CAD1*	2821	813	7.6	90.64
AT2G23200	*CAD2*	2633	806	5.97	90.68
***L. japonicus***					
Lj1g3v4996200	*ANXUR*	2592	863	5.46	95.4
Lj0g3v0115159	*BUPS*	2643	880	5.93	96.29
Lj3g3v3639930	*CURVY1*	2472	823	6.08	90.91
Lj3g3v3639940	*CURVY2*	2121	706	5.98	77.77
Lj1g3v2533770	*FERONIA*	2094	697	5.96	75.96
Lj0g3v0249939	*HERKULES1A*	2514	837	5.41	91.74
Lj3g3v3132890	*HERKULES1B*	1848	615	6.61	67.66
Lj6g3v1641160	*HERKULES2A*	2532	843	5.77	92.66
Lj6g3v1641170	*HERKULES2B*	2532	843	5.77	92.66
Lj2g3v1102970	*MEDOS1*	2676	891	6.75	97.76
Lj2g3v1226730	*MEDOS2*	1542	513	7.59	58.1
Lj2g3v1226740	*MEDOS3*	2466	821	6.93	92.49
Lj2g3v1226750	*MEDOS4*	2277	758	5.49	84.89
Lj0g3v0346559	*THESEUS*	2535	844	5.54	92.59
Lj0g3v0151929	*CAD1*	1554	517	8.87	57.31
Lj2g3v0322770	*CAD2*	2493	830	6.46	92.25
Lj2g3v1902230	*CAD3*	2169	722	8.75	80.67
Lj5g3v1988700	*CAD4*	2535	844	6.98	94.3
***M. truncatula***					
Medtr1g080740	*ANX1*	2607	868	6.15	96.86
Medtr7g115300	*ANX2*	2619	872	5.32	97.04
Medtr8g037700	*BUPS1*	2406	801	5.94	96.47
Medtr4g109010	*CAP1*	3459	1152	6.5	129.96
Medtr4g111925	*FER1*	2106	701	6.5	76.58
Medtr7g073660	*FER2*	2700	899	5.89	97.98
Medtr4g061930	*HERK1A*	2523	840	5.82	93.13
Medtr2g096160	*HERK1B*	2544	847	5.57	92.93
Medtr4g061833	*HERK1C*	2523	840	5.82	93.13
Medtr2g030310	*HERK2*	2628	875	5.89	96.34
Medtr6g015805	*MEDOS1A*	2703	900	6.8	100.32
Medtr5g047120	*MEDOS2A*	2430	809	7.09	92.07
Medtr5g047070	*MEDOS2B*	1707	568	6.23	64.53
Medtr7g015390	*MEDOS3A*	2670	889	6.25	101.42
Medtr7g015550	*MEDOS3B*	2667	888	5.92	100.81
Medtr7g015670	*MEDOS3C*	2679	892	5.75	101.65
Medtr7g015510	*MEDOS3D*	2667	888	5.84	100.38
Medtr7g015240	*MEDOS3E*	2529	842	6.16	96.25
Medtr7g015280	*MEDOS3F*	2577	858	6.44	97.98
Medtr7g015420	*MEDOS3G*	3417	1138	6.02	130.78
Medtr4g052290	*MEDOS3H*	2658	885	7.8	101.29
Medtr7g015250	*MEDOS3I*	2733	910	6.16	103.04
Medtr7g015310	*MEDOS3J*	2622	873	6.08	98.98
Medtr7g015230	*MEDOS3K*	2652	883	6.91	100.7
Medtr7g015320	*MEDOS3L*	2637	878	7.58	99.19
Medtr7g015620	*MEDOS3M*	2064	687	8.81	78.9
Medtr5g047060	*MEDOS4A*	2502	833	5.21	94.17
Medtr5g047110	*MEDOS4B*	2454	817	5.5	92.57
Medtr6g048090	*OG1*	2385	794	6.84	88.69
Medtr1g100110	*OG2*	2445	814	6.57	91.66
Medtr2g080220	*THE1*	2532	843	5.61	92.73
Medtr4g095042	*CAD1*	2556	851	5.85	94.87
Medtr4g095012	*CAD2*	2460	819	6.12	91.27
Medtr4g095032	*CAD3*	2361	786	5.82	87.7
Medtr1g040073	*CAD4*	2256	751	6.23	83.97
Medtr8g467150	*CAD5*	2277	758	7.33	85.16

* Phytozome ID. bp: base pairs. CDS: coding sequence. aa: amino acids. iP: isoelectric point. kDa: kiloDalton.

## Supplementary Materials

The following data are available online at https://www.mdpi.com/2073-4425/11/7/793/s1, Figure S1: Phylogenetic relationship among 1050 CrRLK1L proteins. Figure S2: Unrooted approximately maximum-likelihood phylogenetic tree of the CrRLK1L subfamily proteins in various species. Figure S3: Chromosomal location of *CrRLK1L* genes in various species. Figure S4: Synteny of all *CrRLK1L* genes of *P. vulgaris*, *L. japonicus*, *G. max*, *M. truncatula*, and *A. thaliana* and of the complete genome of each species. Figure S5: Venn diagram of *CrRLK1Ls* genes expressed in nodules of *P. vulgaris*, *L. japonicus*, *G. max*, and *M. truncatula*. Table S1: Oligonucleotides designed for gene-specific detection by RT-qPCR. Table S2: Number of *CrRLK1L* subfamily genes in 62 different species. Table S3. ID list of 1050 *CrRLK1L* genes present in 57 species by clade. Table S4: List of syntenic gene pairs founded by species and between species.

## References

[1] Shiu S.-H., Bleecker A.B. (2001). Receptor-like kinases from Arabidopsis form a monophyletic gene family related to animal receptor kinases. Proc. Natl. Acad. Sci. USA.

[2] Schulze-Muth P., Irmler S., Schröder G., Schröder J. (1996). Novel type of receptor-like protein kinase from a higher plant (*Catharanthus roseus*): cDNA, gene, intramolecular autophosphorylation, and identification of a threonine important for auto- and substrate phosphorylation. J. Biol. Chem..

[3] Boisson-Dernier A., Kessler S.A., Grossniklaus U. (2011). The walls have ears: The role of plant CrRLK1Ls in sensing and transducing extracellular signals. J. Exp. Bot..

[4] Guo H., Li L., Ye H., Yu X., Algreen A., Yin Y. (2009). Three related receptor-like kinases are required for optimal cell elongation in Arabidopsis thaliana. Proc. Natl. Acad. Sci. USA.

[5] DesLauriers S.D., Larsen P.B. (2010). Feronia is a Key Modulator of Brassinosteroid and Ethylene Responsiveness in Arabidopsis Hypocotyls. Mol. Plant.

[6] Yu F., Qian L., Nibau C., Duan Q., Kita D., Levasseur K., Li X., Lu C., Li H., Hou C. (2012). FERONIA receptor kinase pathway suppresses abscisic acid signaling in Arabidopsis by activating ABI2 phosphatase. Proc. Natl. Acad. Sci. USA.

[7] Mao D., Yu F., Li J., Van De Poel B., Tan D., Li J., Liu Y., Li X., Dong M., Chen L. (2015). FERONIA receptor kinase interacts withS-adenosylmethionine synthetase and suppressesS-adenosylmethionine production and ethylene biosynthesis inArabidopsis. Plant Cell Environ..

[8] Liao H., Tang R., Zhang X., Luan S., Yu F. (2017). FERONIA Receptor Kinase at the Crossroads of Hormone Signaling and Stress Responses. Plant Cell Physiol..

[9] Guo H., Nolan T.M., Song G., Liu S., Xie Z., Chen J., Schnable P.S., Walley J.W., Yin Y. (2018). FERONIA Receptor Kinase Contributes to Plant Immunity by Suppressing Jasmonic Acid Signaling in Arabidopsis thaliana. Curr. Biol..

[10] Duan Q., Kita D., Li C., Cheung A.Y., Wu H.-M. (2010). FERONIA receptor-like kinase regulates RHO GTPase signaling of root hair development. Proc. Natl. Acad. Sci. USA.

[11] Kessler S., Shimosato-Asano H., Keinath N.F., Wuest S.E., Ingram G.C., Panstruga R., Grossniklaus U. (2010). Conserved Molecular Components for Pollen Tube Reception and Fungal Invasion. Science.

[12] Shen Q., Bourdais G., Pan H., Robatzek S., Tang D. (2017). Arabidopsis glycosylphosphatidylinositol-anchored protein LLG1 associates with and modulates FLS2 to regulate innate immunity. Proc. Natl. Acad. Sci. USA.

[13] Zhu S., Estévez J.M., Liao H., Zhu Y., Yang T., Li C., Wang Y., Li L., Liu X., Pacheco J.M. (2020). The RALF1–FERONIA Complex Phosphorylates eIF4E1 to Promote Protein Synthesis and Polar Root Hair Growth. Mol. Plant.

[14] Xu G., Chen W., Song L., Chen Q., Zhang H., Liao H., Zhao G., Lin F., Zhou H., Yu F. (2019). Feronia phosphorylates E3 ubiquitin ligase ATL6 to modulate the stability of 14-3-3 proteins in response to the carbon/nitrogen ratio. J. Exp. Bot..

[15] Huck N., Moore J.M., Federer M., Grossniklaus U. (2003). The Arabidopsis mutant feronia disrupts the female gametophytic control of pollen tube reception. Development.

[16] Escobar-Restrepo J.-M., Huck N., Kessler S.A., Gagliardini V., Gheyselinck J., Yang W.-C., Grossniklaus U. (2007). The FERONIA Receptor-like Kinase Mediates Male-Female Interactions during Pollen Tube Reception. Science.

[17] Rotman N., Gourgues M., Guitton A.-E., Faure J.-E., Berger F. (2008). A Dialogue between the Sirène Pathway in Synergids and the Fertilization Independent Seed Pathway in the Central Cell Controls Male Gamete Release during Double Fertilization in Arabidopsis. Mol. Plant.

[18] Ngo Q.A., Vogler H., Lituiev D.S., Nestorova A., Grossniklaus U. (2014). A Calcium Dialog Mediated by the FERONIA Signal Transduction Pathway Controls Plant Sperm Delivery. Dev. Cell.

[19] Kessler S.A., Lindner H., Jones D.S., Grossniklaus U. (2014). Functional analysis of related Cr RLK 1L receptor-like kinases in pollen tube reception. EMBO Rep..

[20] Stegmann M., Monaghan J., Smakowska-Luzan E., Rovenich H., Lehner A., Holton N., Belkhadir Y., Zipfel C. (2017). The receptor kinase FER is a RALF-regulated scaffold controlling plant immune signaling. Science.

[21] Campos W.F., Dressano K., Ceciliato P.H.O., Guerrero-Abad J.C., Silva A.L., Fiori C.S., Canto A.M.D., Bergonci T., Claus L.A.N., Silva-Filho M.C. (2017). Arabidopsis thaliana rapid alkalinization factor 1–mediated root growth inhibition is dependent on calmodulin-like protein 38. J. Biol. Chem..

[22] Yu F., Li J., Huang Y., Liu L., Li D., Chen L., Luan S. (2014). FERONIA Receptor Kinase Controls Seed Size in Arabidopsis thaliana. Mol. Plant.

[23] Zhao C., Zayed O., Yu Z., Jiang W., Zhu P., Hsu C.-C., Zhang L., Tao W.A., Lozano-Durán R., Zhu J. (2018). Leucine-rich repeat extensin proteins regulate plant salt tolerance in Arabidopsis. Proc. Natl. Acad. Sci. USA.

[24] Yang T., Wang L., Li C., Liu Y., Zhu S., Qi Y., Liu X., Lin Q., Luan S., Yu F. (2015). Receptor protein kinase FERONIA controls leaf starch accumulation by interacting with glyceraldehyde-3-phosphate dehydrogenase. Biochem. Biophys. Res. Commun..

[25] Li C., Liu X., Qiang X., Li X., Li X., Zhu S., Wang L., Wang Y., Liao H., Luan S. (2018). EBP1 nuclear accumulation negatively feeds back on FERONIA-mediated RALF1 signaling. PLoS Biol..

[26] Wang L., Yang T., Lin Q., Wang B., Li X., Luan S., Yu F. (2020). Receptor kinase FERONIA regulates flowering time in Arabidopsis. BMC Plant Biol..

[27] Richter J., Ploderer M., Mongelard G., Gutierrez L., Hauser M.-T. (2017). Role of CrRLK1L Cell Wall Sensors HERCULES1 and 2, THESEUS1, and FERONIA in Growth Adaptation Triggered by Heavy Metals and Trace Elements. Front. Plant Sci..

[28] Yang Z., Xing J., Wang L., Liu Y., Qu J., Tan Y., Fu X., Lin Q., Deng H., Yu F. (2020). Mutations of two FERONIA-like receptor genes enhance rice blast resistance without growth penalty. J. Exp. Bot..

[29] Jia M., Ding N., Zhang Q., Xing S., Wei L., Zhao Y., Du P., Mao W., Li J., Li B. (2017). A FERONIA-Like Receptor Kinase Regulates Strawberry (Fragaria × ananassa) Fruit Ripening and Quality Formation. Front. Plant Sci..

[30] Zhu L., Chu L.-C., Liang Y., Zhang X.-Q., Chen L.-Q., Ye D. (2018). The Arabidopsis CrRLK1L protein kinases BUPS1 and BUPS2 are required for normal growth of pollen tubes in the pistil. Plant J..

[31] Boisson-Dernier A., Roy S., Kritsas K., Grobei M.A., Jaciubek M., Schroeder J.I., Grossniklaus U. (2009). Disruption of the pollen-expressed FERONIA homologs ANXUR1 and ANXUR2 triggers pollen tube discharge. Development.

[32] Boisson-Dernier A., Lituiev D.S., Nestorova A., Franck C.M., Thirugnanarajah S., Grossniklaus U. (2013). ANXUR receptor-like kinases coordinate cell wall integrity with growth at the pollen tube tip via NADPH oxidases. PLoS Biol..

[33] Galindo-Trigo S., Blanco-Touriñán N., DeFalco T.A., Wells E.S., Gray J.E., Zipfel C., Smith L.M. (2019). Cr RLK 1L receptor-like kinases HERK 1 and ANJEA are female determinants of pollen tube reception. EMBO Rep..

[34] Hematy K., Höfte H. (2008). Novel receptor kinases involved in growth regulation. Curr. Opin. Plant Biol..

[35] Schoenaers S., Balcerowicz D., Costa A., Vissenberg K. (2017). The Kinase ERULUS Controls Pollen Tube Targeting and Growth in Arabidopsis thaliana. Front. Plant Sci..

[36] Schoenaers S., Balcerowicz D., Breen G., Hill K., Zdanio M., Mouille G., Holman T.J., Oh J., Wilson M.H., Nikonorova N. (2018). The Auxin-Regulated CrRLK1L Kinase ERULUS Controls Cell Wall Composition during Root Hair Tip Growth. Curr. Biol..

[37] Gachomo E.W., Jno Baptiste L., Kefela T., Saidel W.M., Kotchoni S.O. (2014). The Arabidopsis CURVY1 (CVY1) gene encoding a novel receptor-like protein kinase regulates cell morphogenesis, flowering time and seed production. BMC Plant Biol..

[38] Richter J., Watson J.M., Stasnik P., Borowska M., Neuhold J., Berger M., Stolt-Bergner P., Schoft V., Hauser M.-T. (2018). Multiplex mutagenesis of four clustered CrRLK1L with CRISPR/Cas9 exposes their growth regulatory roles in response to metal ions. Sci. Rep..

[39] Haruta M., Sabat G., Stecker K., Minkoff B.B., Sussman M.R. (2014). A Peptide Hormone and Its Receptor. Science.

[40] Ge Z., Bergonci T., Zhao Y., Zou Y., Du S., Liu M.-C., Luo X., Ruan H., García-Valencia L.E., Zhong S. (2017). Arabidopsispollen tube integrity and sperm release are regulated by RALF-mediated signaling. Science.

[41] Wu J., Kurten E.L., Monshausen G., Hummel G.M., Gilroy S., Baldwin I.T. (2007). NaRALF, a peptide signal essential for the regulation of root hair tip apoplastic pH inNicotiana attenuata, is required for root hair development and plant growth in native soils. Plant J..

[42] Covey P.A., Subbaiah C.C., Parsons R.L., Pearce G., Lay F.T., Anderson M.A., Ryan C.A., Bedinger P.A. (2010). A pollen-specific RALF from tomato that regulates pollen tube elongation. Plant Physiol..

[43] Cao J., Shi F. (2012). Evolution of the RALF Gene Family in Plants: Gene Duplication and Selection Patterns. Evol. Bioinform..

[44] Campbell L., Turner S.R. (2017). A Comprehensive Analysis of RALF Proteins in Green Plants Suggests There Are Two Distinct Functional Groups. Front. Plant Sci..

[45] Combier J.-P., Küster H., Journet E.-P., Hohnjec N., Gamas P., Niebel A. (2008). Evidence for the Involvement in Nodulation of the Two Small Putative Regulatory Peptide-Encoding GenesMtRALFL1andMtDVL1. Mol. Plant Microbe Interact..

[46] Doyle J.J., Luckow M.A. (2003). The Rest of the Iceberg. Legume Diversity and Evolution in a Phylogenetic Context. Plant Physiol..

[47] Ferguson B., Mens C., Hastwell A., Zhang M., Su H., Jones C.M., Chu X., Gresshoff P.M. (2018). Legume nodulation: The host controls the party. Plant Cell Environ..

[48] Goodstein D., Shu S., Howson R., Neupane R., Hayes R., Fazo J., Mitros T., Dirks W., Hellsten U., Putnam N.H. (2011). Phytozome: A comparative platform for green plant genomics. Nucleic Acids Res..

[49] Mun T., Bachmann A., Gupta V., Stougaard J., Andersen S.U. (2016). Lotus Base: An integrated information portal for the model legume Lotus japonicus. Sci. Rep..

[50] El-Gebali S., Mistry J., Bateman A., Eddy S.R., Luciani A., Potter S.C., Qureshi M., Richardson L.J., Salazar G.A., Smart A. (2019). The Pfam protein families database in 2019. Nucleic Acids Res..

[51] Edgar R.C. (2004). MUSCLE: Multiple sequence alignment with high accuracy and high throughput. Nucleic Acids Res..

[52] Larsson A. (2014). AliView: A fast and lightweight alignment viewer and editor for large datasets. Bioinformatics.

[53] Guindon S., Lefort V., Anisimova M., Hordijk W., Gascuel O., Dufayard J.-F. (2010). New Algorithms and Methods to Estimate Maximum-Likelihood Phylogenies: Assessing the Performance of PhyML 3.0. Syst. Biol..

[54] Nguyen L.-T., Schmidt H.A., Von Haeseler A., Minh B.Q. (2014). IQ-TREE: A Fast and Effective Stochastic Algorithm for Estimating Maximum-Likelihood Phylogenies. Mol. Biol. Evol..

[55] Kumar S., Stecher G., Tamura K. (2016). MEGA7: Molecular Evolutionary Genetics Analysis version 7.0 for bigger datasets. Mol. Biol. Evol..

[56] Bailey T.L., Bodén M., Buske F.A., Frith M., Grant C.E., Clementi L., Ren J., Li W.W., Noble W.S. (2009). MEME SUITE: Tools for motif discovery and searching. Nucleic Acids Res..

[57] Gasteiger E., Hoogland C., Gattiker A., Duvaud S., Wilkins M.R., Appel R.D., Bairoch A. (2005). The Proteomics Protocols Handbook.

[58] Wolfe D., Dudek S., Ritchie M., Pendergrass S.A. (2013). Visualizing genomic information across chromosomes with PhenoGram. BioData Min..

[59] Wang Y., Tang H., DeBarry J.D., Tan X., Li J., Wang X., Lee T.-H., Jin H., Marler B., Guo H. (2012). MCScanX: A toolkit for detection and evolutionary analysis of gene synteny and collinearity. Nucleic Acids Res..

[60] O’Rourke J.A., Iniguez L.P., Fu F., Bucciarelli B., Miller S.S., Jackson S.A., McClean P.E., Li J., Dai X., Zhao P.X. (2014). An RNA-Seq based gene expression atlas of the common bean. BMC Genom..

[61] Benedito V.A., Torres-Jerez I., Murray J.D., Andriankaja A., Allen S., Kakar K., Wandrey M., Verdier J., Zuber H., Ott T. (2008). A gene expression atlas of the model legume Medicago truncatula. Plant J..

[62] He J., Benedito V.A., Wang M., Murray J.D., Zhao P.X., Tang Y., Udvardi M.K. (2009). The Medicago truncatula gene expression atlas web server. BMC Bioinform..

[63] Schmid M., Davison T.S., Henz S.R., Pape U.J., Demar M., Vingron M., Schölkopf B., Weigel D., Lohmann J.U. (2005). A gene expression map of Arabidopsis thaliana development. Nat. Genet..

[64] Libault M., Farmer A., Joshi T., Takahashi K., Langley R.J., Franklin L.D., He J., Xu D., May G., Stacey G. (2010). An integrated transcriptome atlas of the crop model Glycine max, and its use in comparative analyses in plants. Plant J..

[65] Ortiz-Ramírez C., Hernandez-Coronado M., Thamm A., Catarino B., Wang M., Dolan L., Feijó J.A.A., Becker J.D.D. (2016). A Transcriptome Atlas of Physcomitrella patens Provides Insights into the Evolution and Development of Land Plants. Mol. Plant.

[66] Warnes G.R., Bolker B., Huber W., Lumley T., Maechler M., Magnusson A., Moeller S. Gplots: Various R Programming Tools for Plotting Data.

[67] Islas T., Guillén G., Alvarado-Affantranger X., Lara-Flores M., Sánchez F., Villanueva M.A. (2011). PvRACK1 Loss-of-Function Impairs Cell Expansion and Morphogenesis in *Phaseolus vulgaris* L. Root Nodules. Mol. Plant-Microbe Interact..

[68] Nguyen Q.-N., Lee Y.-S., Cho L.-H., Jeong H.-J., An G., Jung K.-H. (2014). Genome-wide identification and analysis of Catharanthus roseus RLK1-like kinases in rice. Planta.

[69] Niu E., Cai C., Zheng Y., Shang X., Fang L., Guo W. (2016). Genome-wide analysis of CrRLK1L gene family in Gossypium and identification of candidate CrRLK1L genes related to fiber development. Mol. Genet. Genom..

[70] Kou X., Qi K., Qiao X., Yin H., Liu X., Zhang S., Wu J. (2017). Evolution, expression analysis, and functional verification of Catharanthus roseus RLK1-like kinase (CrRLK1L) family proteins in pear (*Pyrus bretchneideri*). Genomic.

[71] Morris J.L., Puttick M.N., Clark J.W., Edwards D., Kenrick P., Pressel S., Wellman C.H., Yang Z., Schneider H., Donoghue P.C.J. (2018). The timescale of early land plant evolution. Proc. Natl. Acad. Sci. USA.

[72] Niklas K.J., Kutschera U. (2009). The evolution of the land plant life cycle. New Phytol..

[73] Chang C.-C., Chen H.-L., Li W.-H., Chaw S.-M. (2004). Dating the monocot-dicot divergence and the origin of core eudicots using whole chloroplast genomes. J. Mol. Evol..

[74] Jiao X., Leebens-Mack J., Ayyampalayam S., Bowers J., McKain M.R., McNeal J.R., Rolf M., Ruzicka D.R., Wafula E.K., Wickett N.J. (2012). A genome triplication associated with early diversification of the core eudicots. Genome Biol..

[75] Lynch M., Conery J.S. (2000). The Evolutionary Fate and Consequences of Duplicate Genes. Science.

[76] Coghlan A., Eichler E.E., Oliver S.G., Paterson A.H., Stein L. (2005). Chromosome evolution in eukaryotes: A multi-kingdom perspective. Trends Genet..

[77] Bowers J., Chapman B., Rong J., Paterson A.H. (2003). Unravelling angiosperm genome evolution by phylogenetic analysis of chromosomal duplication events. Nature.

[78] Freeling M. (2009). Bias in Plant Gene Content Following Different Sorts of Duplication: Tandem, Whole-Genome, Segmental, or by Transposition. Annu. Rev. Plant Biol..

[79] Wang Y., Wang X., Tang H., Tan X., Ficklin S.P., Feltus F.A., Paterson A.H. (2011). Modes of Gene Duplication Contribute Differently to Genetic Novelty and Redundancy, but Show Parallels across Divergent Angiosperms. PLoS ONE.

[80] Jami S.K., Clark G.B., Ayele B.T., Ashe P., Kirti P.B. (2012). Genome-wide Comparative Analysis of Annexin Superfamily in Plants. PLoS ONE.

[81] Carle-Urioste J.C., Brendel V., Walbot V. (1997). A combinatorial role for exon, intron and splice site sequences in splicing in maize. Plant J..

[82] Mattick J.S., Gagen M.J. (2001). The evolution of controlled multitasked gene networks: The role of introns and other noncoding RNAs in the development of complex organisms. Mol. Biol. Evol..

[83] Schwartz S., Meshorer E., Ast G. (2009). Chromatin organization marks exon-intron structure. Nat. Struct. Mol. Biol..

[84] Zhu L., Zhang Y., Zhang W., Yang S., Chen J.-Q., Tian D. (2009). Patterns of exon-intron architecture variation of genes in eukaryotic genomes. BMC Genom..

[85] Kiraga J., Mackiewicz P., Mackiewicz D., Kowalczuk M., Biecek P., Polak N., Smolarczyk K., Dudek M.R., Cebrat S. (2007). The relationships between the isoelectric point and: Length of proteins, taxonomy and ecology of organisms. BMC Genom..

[86] Doyle J.J., Egan A.N. (2009). Dating the origins of polyploidy events. New Phytol..

[87] Zhao T., Schranz M.E. (2017). Network approaches for plant phylogenomic synteny analysis. Curr. Opin. Plant Biol..

[88] Schwartz R., Ting C.S., King J. (2001). Whole Proteome pI Values Correlate with Subcellular Localizations of Proteins for Organisms within the Three Domains of Life. Genome Res..

[89] Weiller G.F., Caraux G., Silvester N. (2004). The modal distribution of protein isoelectric points reflects amino acid properties rather than sequence evolution. Proteomics.

[90] Yoder J.B. (2016). Understanding the coevolutionary dynamics of mutualism with population genomics. Am. J. Bot..

[91] De Moura G.G.D., Remigi P., Masson-Boivin C., Capela D. (2020). Experimental Evolution of Legume Symbionts: What Have We Learnt?. Genes.

[92] De Mita S., Streng A., Bisseling T., Geurts R. (2013). Evolution of a symbiotic receptor through gene duplications in the legume-rhizobium mutualism. New Phytol..

[93] Patriarca E.J., Tatè R., Ferraioli S., Iaccarino M. (2004). Organogenesis of Legume Root Nodules. Int. Rev. Cytol..

[94] Monahan-Giovanelli H., Pinedo C.A., Gage D.J. (2006). Architecture of Infection Thread Networks in Developing Root Nodules Induced by the Symbiotic Bacterium Sinorhizobium meliloti on Medicago truncatula. Plant Physiol..

[95] Ferguson B., Indrasumunar A., Hayashi S., Lin M.-H., Lin Y.-H., Reid D.E., Gresshoff P.M. (2010). Molecular Analysis of Legume Nodule Development and Autoregulation. J. Integr. Plant Biol..

[96] Popp C., Ott T. (2011). Regulation of signal transduction and bacterial infection during root nodule symbiosis. Curr. Opin. Plant Biol..

[97] Xiao T.T., Schilderink S., Moling S., Deinum E.E., Kondorosi E., Franssen H., Kulikova O., Niebel A., Bisseling T. (2014). Fate map of Medicago truncatula root nodules. Development.

[98] Limpens E., Ivanov S., Van Esse G.W., Voets G., Fedorova E., Bisseling T. (2009). Medicago N2-Fixing Symbiosomes Acquire the Endocytic Identity Marker Rab7 but Delay the Acquisition of Vacuolar Identity. Plant Cell.

[99] Gavrin A., Chiasson D., Ovchinnikova E., Kaiser B.N., Bisseling T., Fedorova E.E. (2016). VAMP721a and VAMP721d are important for pectin dynamics and release of bacteria in soybean nodules. New Phytol..

[100] Cheung A.Y., Wu H.-M. (2011). THESEUS 1, FERONIA and relatives: A family of cell wall-sensing receptor kinases?. Curr. Opin. Plant Biol..

[101] Mecchia M.A., Santos-Fernandez G., Duss N.N., Somoza S.C., Boisson-Dernier A., Gagliardini V., Martinez-Bernardini A., Fabrice T., Ringli C., Muschietti J. (2017). RALF4/19 peptides interact with LRX proteins to control pollen tube growth inArabidopsis. Science.

[102] Vogler H., Santos-Fernandez G., Mecchia M.A., Grossniklaus U. (2019). To preserve or to destroy, that is the question: The role of the cell wall integrity pathway in pollen tube growth. Curr. Opin. Plant Biol..

[103] Kuppusamy K.T., Ivashuta S., Bucciarelli B., Vance C.P., Gantt J.S., VandenBosch K.A. (2009). Knockdown of CELL DIVISION CYCLE16 reveals an inverse relationship between lateral root and nodule numbers and a link to auxin in Medicago truncatula. Plant Physiol..

[104] Suzaki T., Yano K., Ito M., Umehara Y., Suganuma N., Kawaguchi M. (2012). Positive and negative regulation of cortical cell division during root nodule development in Lotus japonicus is accompanied by auxin response. Development.

[105] Guan D., Stacey N., Liu C.-W., Wen J., Mysore K.S., Torres-Jerez I., Vernié T., Tadege M., Zhou C., Wang Z.-Y. (2013). Rhizobial infection is associated with the development of peripheral vasculature in nodules of Medicago truncatula. Plant Physiol..

[106] Sogawa A., Yamazaki A., Yamasaki H., Komi M., Manabe T., Tajima S., Hayashi M., Nomura M. (2019). SNARE Proteins LjVAMP72a and LjVAMP72b Are Required for Root Symbiosis and Root Hair Formation in Lotus japonicus. Front. Plant Sci..

[107] Foucher F., Kondorosi E. (2000). Cell cycle regulation in the course of nodule organogenesis in Medicago. Plant Mol. Biol..

[108] Zipfel C., Oldroyd G.E.D. (2017). Plant signalling in symbiosis and immunity. Nature.

[109] Mergaert P., Uchiumi T., Alunni B., Evanno G., Cheron A., Catrice O., Mausset A.-E., Barloy-Hubler F., Galibert F., Kondorosi A. (2006). Eukaryotic control on bacterial cell cycle and differentiation in the Rhizobium-legume symbiosis. Proc. Natl. Acad. Sci. USA.

[110] Ott T., Sullivan J., James E.K., Flemetakis E., Günther C.S., Gibon Y., Ronson C., Udvardi M.K. (2009). Absence of Symbiotic Leghemoglobins Alters Bacteroid and Plant Cell Differentiation During Development ofLotus japonicusRoot Nodules. Mol. Plant-Microbe Interact..

[111] Wang L., Rubio M.C., Xin X., Zhang B., Fan Q., Wang Q., Ning G., Becana M., Duanmu D. (2019). CRISPR/Cas9 knockout of leghemoglobin genes in Lotus japonicus uncovers their synergistic roles in symbiotic nitrogen fixation. New Phytol..

[112] Torres M.A., Jones J.D., Dangl J.L. (2005). Pathogen-induced, NADPH oxidase–derived reactive oxygen intermediates suppress spread of cell death in Arabidopsis thaliana. Nat. Genet..

[113] Lohar D.P., Haridas S., VandenBosch K.A., Gantt J.S. (2006). A transient decrease in reactive oxygen species in roots leads to root hair deformation in the legume-rhizobia symbiosis. New Phytol..

[114] Marino D., Andrio E., Danchin E.G.J., Oger E., Gucciardo S., Lambert A., Puppo A., Pauly N. (2010). A Medicago truncatula NADPH oxidase is involved in symbiotic nodule functioning. New Phytol..

[115] Montiel J., Nava N., Cárdenas L., Sanchez-Lopez R., Arthikala M.-K., Santana O., Sánchez F., Quinto C. (2012). A Phaseolus vulgaris NADPH Oxidase Gene is Required for Root Infection by Rhizobia. Plant Cell Physiol..

[116] Arthikala M.-K., Montiel J., Sanchez-Lopez R., Nava N., Cárdenas L., Quinto C. (2017). Respiratory Burst Oxidase Homolog Gene A Is Crucial for Rhizobium Infection and Nodule Maturation and Function in Common Bean. Front. Plant Sci..

[117] Montiel J., Fonseca-García C., Quinto C. (2018). Phylogeny and Expression of NADPH Oxidases during Symbiotic Nodule Formation. Agriculture.

[118] Montiel J., Arthikala M.-K., Cardenas L., Quinto C. (2016). Legume NADPH Oxidases Have Crucial Roles at Different Stages of Nodulation. Int. J. Mol. Sci..

[119] Khadri M., Tejera N.A., Plá C.L. (2006). Alleviation of Salt Stress in Common Bean (*Phaseolus vulgaris*) by Exogenous Abscisic Acid Supply. J. Plant Growth Regul..

[120] Ding Y., Kaló P., Yendrek C., Sun J., Liang Y., Marsh J.F., Harris J.M., Oldroyd G.E.D. (2008). Abscisic Acid Coordinates Nod Factor and Cytokinin Signaling during the Regulation of Nodulation in Medicago truncatula. Plant Cell.

[121] Liang Y., Mitchell D.M., Harris J.M. (2007). Abscisic acid rescues the root meristem defects of the Medicago truncatula latd mutant. Dev. Biol..

[122] Biswas B., Chan P.K., Gresshoff P.M. (2009). A Novel ABA Insensitive Mutant of Lotus japonicus with a Wilty Phenotype Displays Unaltered Nodulation Regulation. Mol. Plant.

[123] Hunter W.J. (2001). Influence of Root-Applied Epibrassinolide and Carbenoxolone on the Nodulation and Growth of Soybean (*Glycine max* L.) Seedlings. J. Agron. Crop Sci..

[124] Ferguson B., Ross J.J., Reid J.B. (2005). Nodulation Phenotypes of Gibberellin and Brassinosteroid Mutants of Pea1. Plant Physiol..

[125] Vardhini B.V., Rao S.S.R. (1999). Effect of brassionosteriods on nodulation and nitrogenase activity in groundnut (*Arachis hypogaea* L.). Plant Growth Regul..

[126] Upreti K., Murti G. (2004). Effects of Brassmosteroids on Growth, Nodulation, Phytohormone Content and Nitrogenase Activity in French Bean Under Water Stress. Biol. Plant..

[127] Sun J., Cardoza V., Mitchell D.M., Bright L., Oldroyd G., Harris J.M. (2006). Crosstalk between jasmonic acid, ethylene and Nod factor signaling allows integration of diverse inputs for regulation of nodulation. Plant J..

[128] Nakagawa T., Kawaguchi M. (2006). Shoot-applied MeJA Suppresses Root Nodulation in Lotus japonicus. Plant Cell Physiol..

[129] Suzuki A., Suriyagoda L., Shigeyama T., Tominaga A., Sasaki M., Hiratsuka Y., Yoshinaga A., Arima S., Agarie S., Sakai T. (2011). Lotus japonicus nodulation is photomorphogenetically controlled by sensing the red/far red (R/FR) ratio through jasmonic acid (JA) signaling. Proc. Natl. Acad. Sci. USA.

[130] Guinel F.C., Geil R.D. (2002). A model for the development of the rhizobial and arbuscular mycorrhizal symbioses in legumes and its use to understand the roles of ethylene in the establishment of these two symbioses. Can. J. Bot..

[131] Gresshoff P.M., Lohar D., Chan P.-K., Biswas B., Jiang Q., Reid D.E., Ferguson B., Stacey G. (2009). Genetic analysis of ethylene regulation of legume nodulation. Plant Signal. Behav..

