# Supplementary material for: Genome-Wide Identification of the CrRLK1L Subfamily and Comparative Analysis of Its Role in the Legume-Rhizobia Symbiosis
Source: Genes (Basel). 2020 Jul 14;11(7):793. doi: 10.3390/genes11070793 (PMC7397338; doi:10.3390/genes11070793)
Supplement: Supplementary file 1 [file genes-11-00793-s001.zip › Supplementary Material/Supplementary Material.pdf]

Supplementary Material

Genome-wide identification of CrRLK1L subfamily genes in diverse species and comparative analysis of their roles in the legume–rhizobia symbiosis

Jorge Solis-Miranda, Citlali Fonseca-Garcia, Nava Noreide, Ronal Pacheco, and Carmen Quinto,*

Departamento de Biología Molecular de Plantas, Instituto de Biotecnología, Universidad Nacional Autónoma de México, Avenida Universidad 2001, Colonia Chamilpa, Cuernavaca, Morelos 62210, Mexico; jsolis@ibt.unam.mx (J.S.-M), fonsecac@ibt.unam.mx (C.F.-G), noreide@ibt.unam.mx (N.N), ronal.pacheco@mail.ibt.unam.mx (R.P.),

*Correspondence: quinto@ibt.unam.mx (C.Q.)

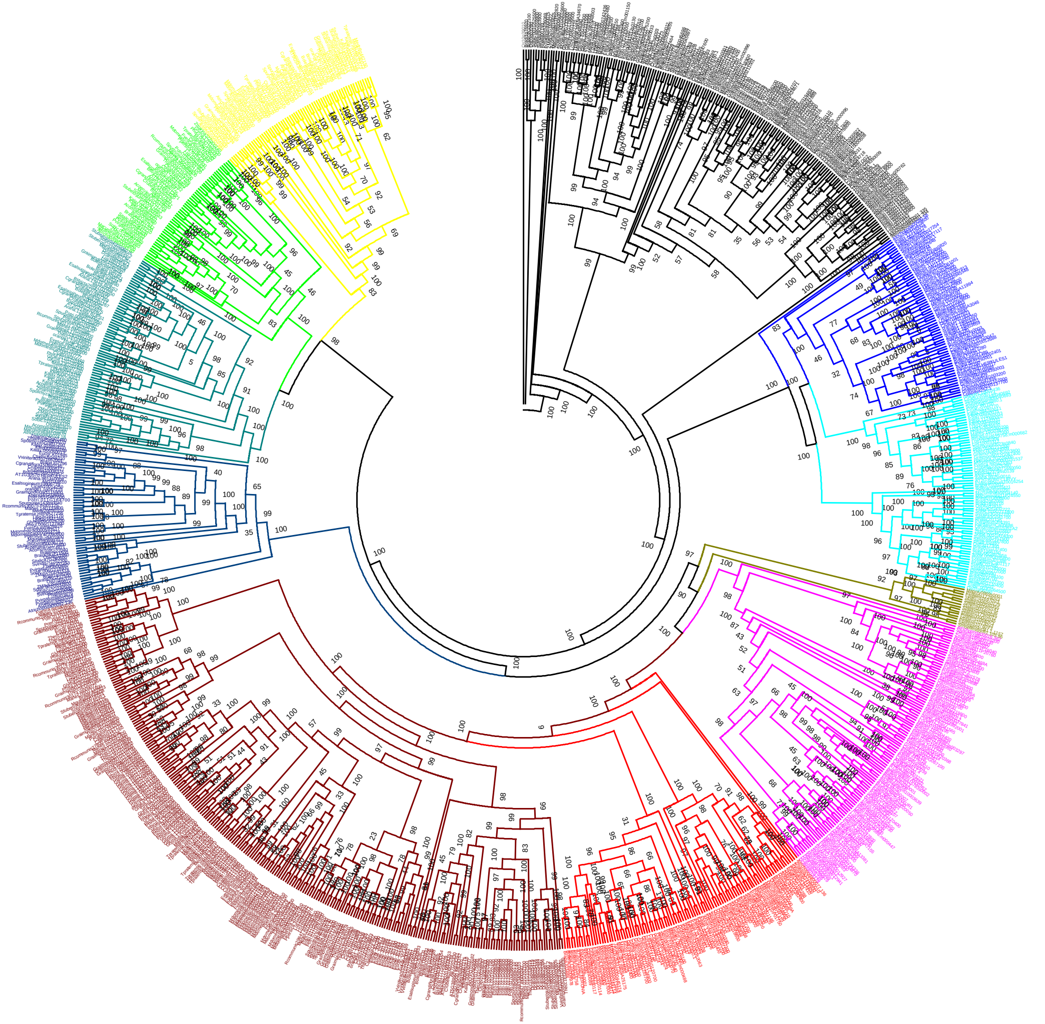

Figure S1: Phylogenetic relationship among 1050 CrRLK1L proteins. High-resolution unrooted approximately maximum-likelihood phylogenetic tree inferred from the 1,050 CrRLK1L proteins present in 57 plant species. The clades, indicated in different colors, are named based on the *A. thaliana* CrRLK1L names. The CADMUS clade contains uncharacterized CrRLK1Ls from *A. thaliana*, and the TINIA clade corresponds to a clade formed only with the CrRLK1L proteins of *S. moellendorffii*, *S. fallax*, *P. patens*, and *M. polymorpha*. The phylogenetic tree was constructed using IQ-TREE software with the JTT+F+R10 substitution model with 1,000 bootstrap iterations. Gene names are highlighted in colors as follows: *ANX* in purple, *BUPS* in green, *CAD* in black, *CAP* in lime, *CRV* in blue, *FER* in red, , *HERK1* (*ANJ*) in blue, *HERK2* in dark blue, *MEDOS* in brown, and *THE* in gray.

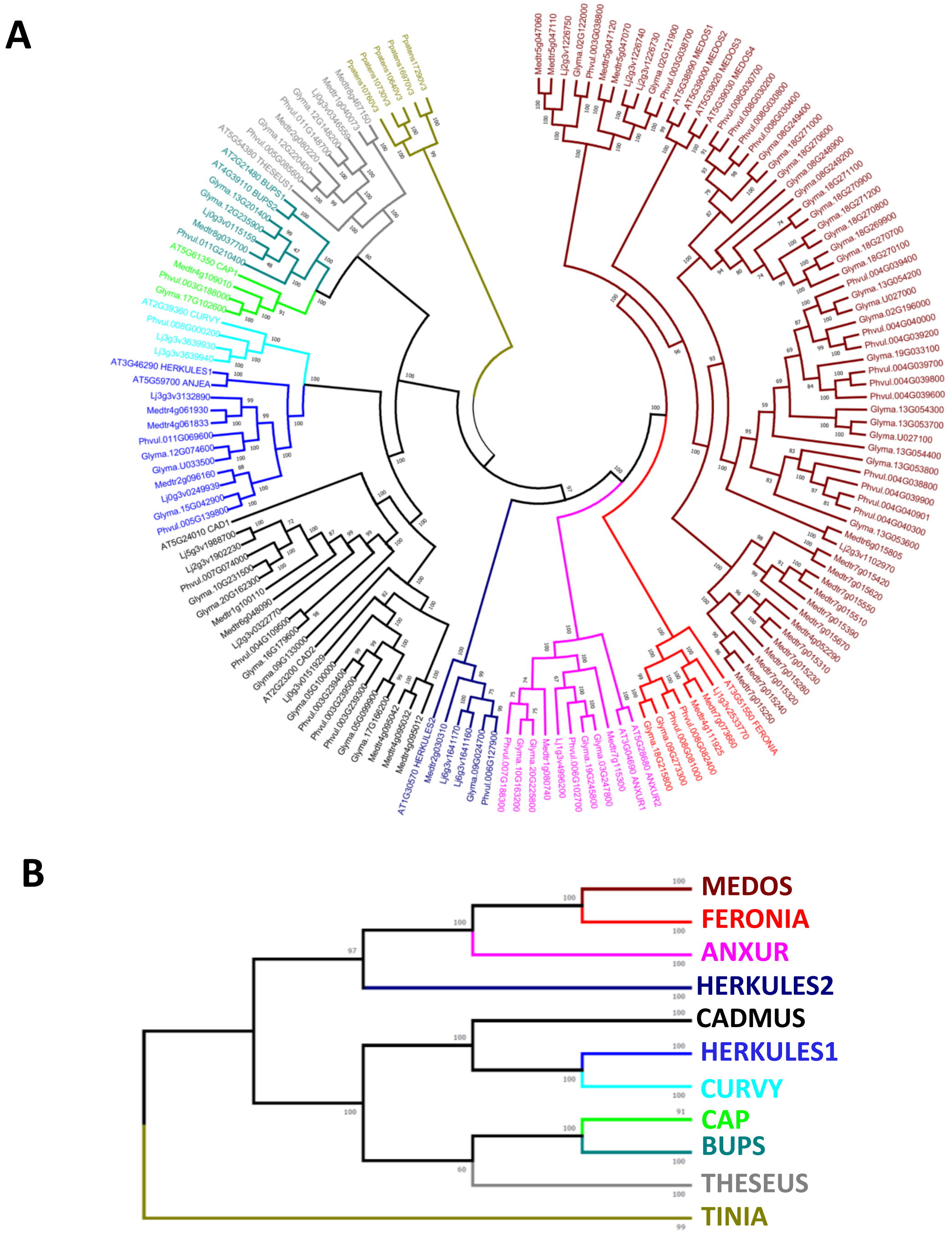

Figure S2. Unrooted approximately maximum-likelihood phylogenetic tree of the CrRLK1L subfamily proteins in various species. A) Inferred phylogenetic tree of 155 CrRLK1L proteins from *P. vulgaris*, *L. japonicus*, *M. truncatula*, *G. max*, *A. thaliana*, and *P. patens*. **B)** Simplified dendrogram representing the 11 clades of the inferred phylogenetic tree in A. Local support values correspond to those in A. Gene names are highlighted in colors as follows: *ANX* in purple, *BUPS* in green, *CAD* in black, *CAP* in lime, *CRV* in blue, *FER* in red, , *HERK1* (*ANJ*) in blue, *HERK2* in dark blue, *MEDOS* in brown, and *THE* in gray.

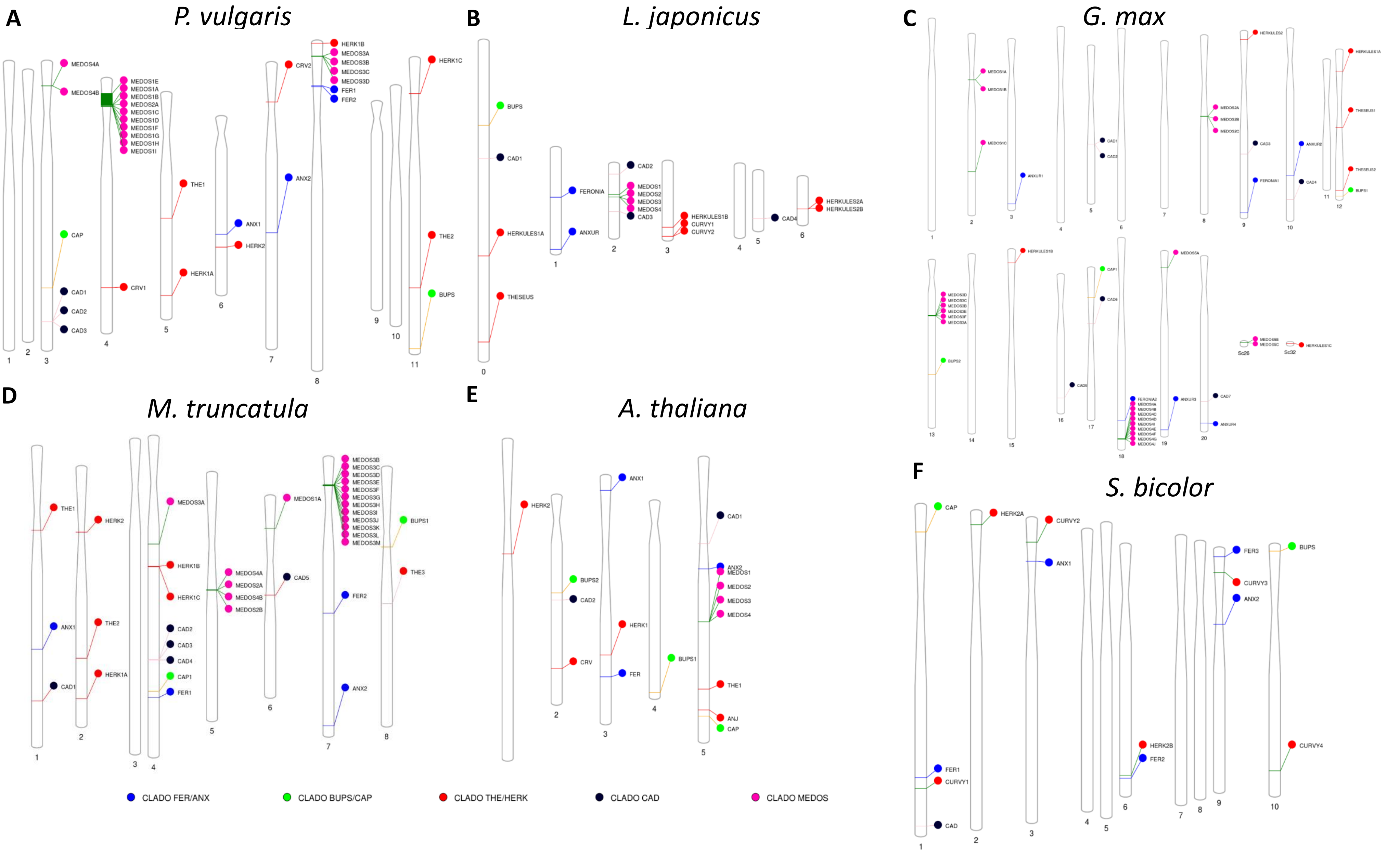
A
P. vulgaris
MEDOS4A
MEDOS4B
MEDOS1E
MEDOS1A
MEDOS1B
MEDOS2A
MEDOS1C
MEDOS1D
MEDOS1F
MEDOS1G
MEDOS1H
MEDOS1I
THE1
HERK1A
ANX1
HERK2
CRV2
ANX2
HERK1B
MEDOS3A
MEDOS3B
MEDOS3C
MEDOS3D
FER1
FER2
HERK1C
THE2
BUPS
CAP
CAD1
CAD2
CAD3
CRV1
B
L. japonicus
BUPS
CAD1
HERKULES1A
THESEUS
FERONIA
ANXUR
CAD2
MEDOS1
MEDOS2
MEDOS3
MEDOS4
CAD3
HERKULES1B
CURVY1
CURVY2
CAD4
HERKULES2A
HERKULES2B
C
G. max
D
M. truncatula
THE1
ANX1
CAD1
HERK2
THE2
HERK1A
MEDOS3A
HERK1B
HERK1C
CAD2
CAD3
CAD4
CAP1
FER1
MEDOS4A
MEDOS2A
MEDOS4B
MEDOS2B
MEDOS1A
CAD5
MEDOS3B
MEDOS3C
MEDOS3D
MEDOS3E
MEDOS3F
MEDOS3G
MEDOS3H
MEDOS3I
MEDOS3J
MEDOS3K
MEDOS3L
MEDOS3M
FER2
ANX2
BUPS1
THE3
E
A. thaliana
HERK2
BUPS2
CAD2
CRV
ANX1
HERK1
FER
BUPS1
CAD1
ANX2
MEDOS1
MEDOS2
MEDOS3
MEDOS4
THE1
ANJ
CAP
F
S. bicolor
CAP
FER1
CURVY1
CAD
HERK2A
CURVY2
ANX1
HERK2B
FER2
FER3
CURVY3
ANX2
BUPS
CURVY4
CLADO FER/ANX
CLADO BUPS/CAP
CLADO THE/HERK
CLADO CAD
CLADO MEDOS

Figure S3. Chromosomal location of *CrRLK1L* genes in various species. Chromosomal location of *CrRLK1L* genes in **A)** *P. vulgaris*, **B)** *L. japonicus*, **C)** *G. max*, **D)** *M. truncatula*, **E)** *A. thaliana*, and **F)** *S. bicolor*. The colored dots and lines indicate the chromosomal location of the different genes.

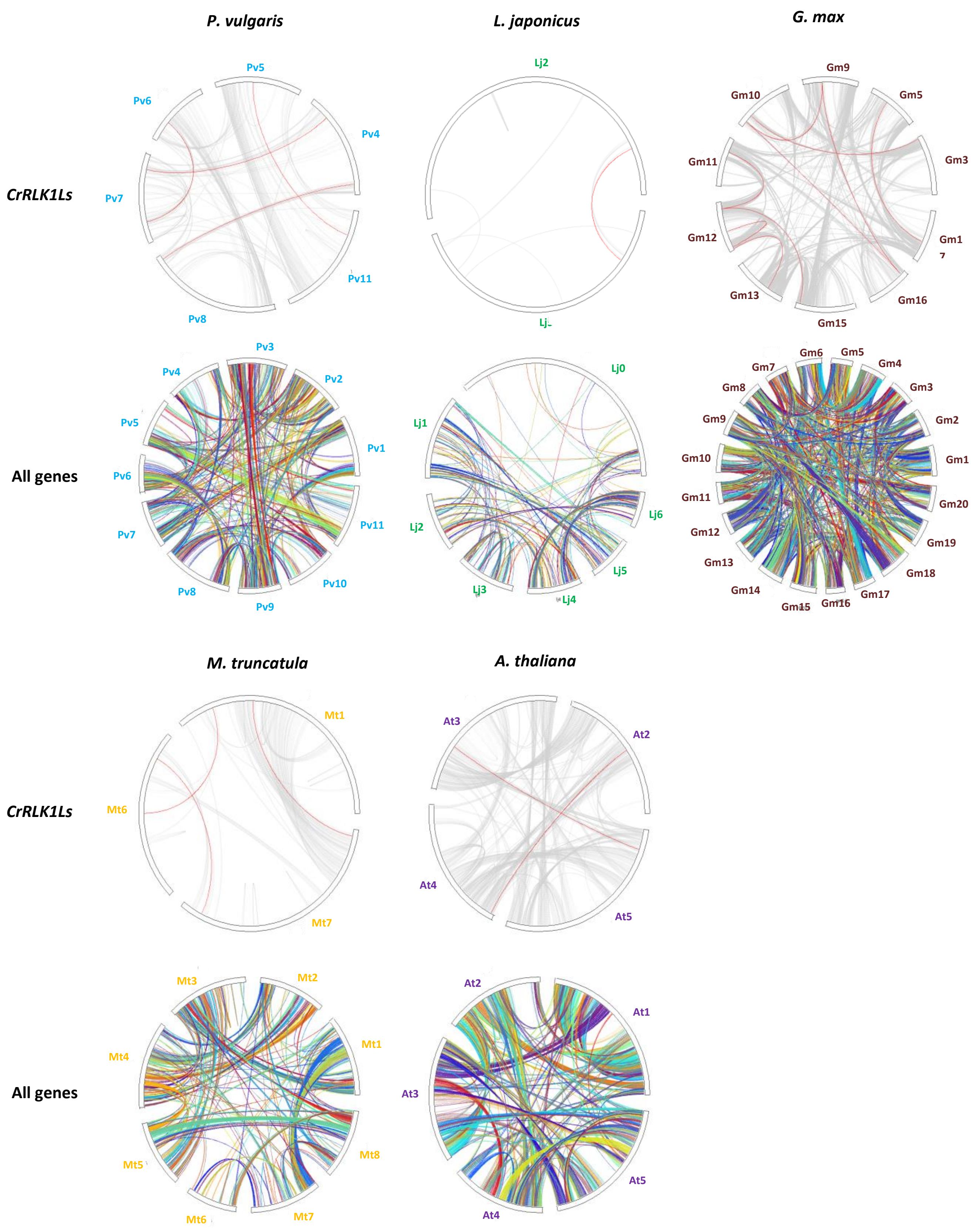
P. vulgaris
L. japonicus
G. max
CrRLK1Ls
Pv5
Pv6
Pv4
Pv7
Pv11
Pv8
Lj2
Gm9
Gm10
Gm5
Gm3
Gm11
Gm12
Gm1
Gm13
Gm16
Gm15
All genes
Pv3
Pv4
Pv2
Pv5
Pv1
Pv6
Pv11
Pv7
Pv8
Pv10
Pv9
Lj0
Lj1
Lj2
Lj6
Lj5
Lj3
Lj4
Gm6
Gm5
Gm7
Gm4
Gm8
Gm3
Gm9
Gm2
Gm10
Gm1
Gm11
Gm20
Gm12
Gm19
Gm13
Gm18
Gm14
Gm17
Gm15
Gm16
M. truncatula
A. thaliana
CrRLK1Ls
Mt1
Mt6
Mt7
At3
At2
At4
At5
All genes
Mt3
Mt2
Mt4
Mt1
Mt5
Mt8
Mt6
Mt7
At2
At1
At3
At5
At4

Figure S4. Synteny of all *CrRLK1L* genes of *P. vulgaris*, *L. japonicus*, *G. max*, *M. truncatula*, and *A. thaliana* and of the complete genome of each species. First and third rows, the *CrRLK1L* syntenic genes are represented as connected red lines. Only chromosomes containing syntenic genes are outlined. Second and fourth rows, all the syntenic genes present in the genome of each species are represented as connected colored lines.

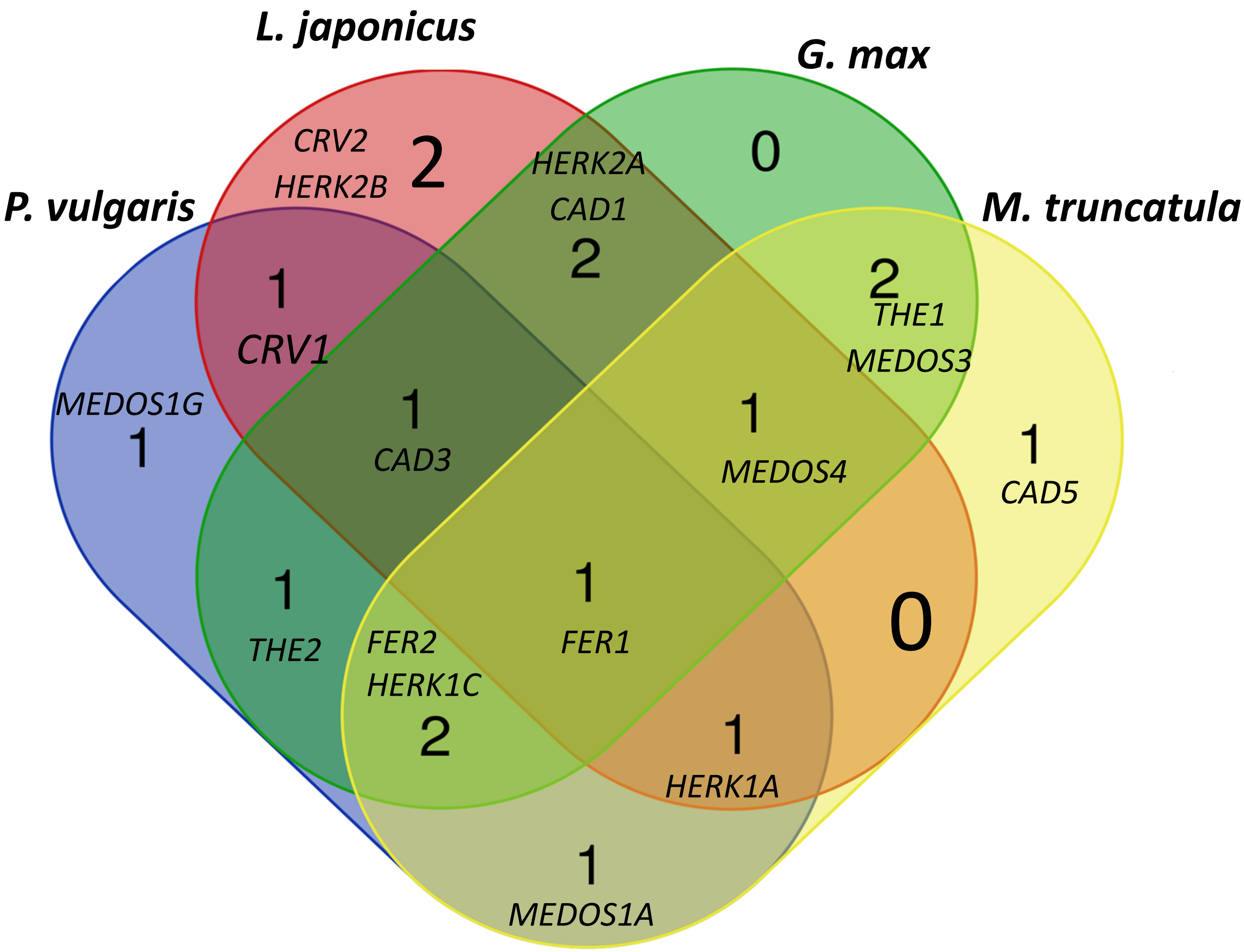

Figure S5. Venn diagram of *CrRLK1Ls* genes expressed in nodules of *P. vulgaris*, *L. japonicus*, *G. max*, and *M. truncatula*. Venn diagram representing the number and names of the genes expressed in nodules. Each of the four ovals represents a set of nodule expressed genes on each legume. Names and numbers in the interconnections represent the nodule-expressed genes shared between the four legumes according to data from PvGEA, Lotus Base, MtGEA, and BAR.
